# Identification of QTL regions and candidate genes for growth and feed efficiency in broilers

**DOI:** 10.1186/s12711-021-00608-3

**Published:** 2021-02-06

**Authors:** Wei Li, Maiqing Zheng, Guiping Zhao, Jie Wang, Jie Liu, Shunli Wang, Furong Feng, Dawei Liu, Dan Zhu, Qinghe Li, Liping Guo, Yuming Guo, Ranran Liu, Jie Wen

**Affiliations:** 1 State Key Laboratory of Animal Nutrition; Key Laboratory of Animal (Poultry) Genetics Breeding and Reproduction, Ministry of Agriculture, Institute of Animal Sciences, Chinese Academy of Agricultural Sciences, Beijing, 100193 China; 2 College of Animal Science and Technology, China Agricultural University, Beijing, 100193 China; 3 Foshan Gaoming Xinguang Agricultural and Animal Industrials Corporation, Foshan, 528515 China

## Abstract

**Background:**

Feed accounts for about 70% of the total cost of poultry meat production. Residual feed intake (RFI) has become the preferred measure of feed efficiency because it is phenotypically independent of growth rate and body weight. In this study, our aim was to estimate genetic parameters and identify quantitative trait loci (QTL) for feed efficiency in 3314 purebred broilers using a genome-wide association study. Broilers were genotyped using a custom 55 K single nucleotide polymorphism (SNP) array.

**Results:**

Estimates of genomic heritability for seven growth and feed efficiency traits, including body weight at 28 days of age (BW28), BW42, average daily feed intake (ADFI), RFI, and RFI adjusted for weight of abdominal fat (RFIa), ranged from 0.12 to 0.26. Eleven genome-wide significant SNPs and 15 suggestively significant SNPs were detected, of which 19 clustered around two genomic regions. A region on chromosome 16 (2.34–2.66 Mb) was associated with both BW28 and BW42, and the most significant SNP in this region, AX_101003762, accounted for 7.6% of the genetic variance of BW28. The other region, on chromosome 1 (91.27–92.43 Mb) was associated with RFI and ADFI, and contains the *NSUN3* and *EPHA6* as candidate genes. The most significant SNP in this region, AX_172588157, accounted for 4.4% of the genetic variance of RFI. In addition, a genomic region containing the gene *AGK* on chromosome 1 was found to be associated with RFIa. The *NSUN3* and *AGK* genes were found to be differentially expressed in breast muscle, thigh muscle, and abdominal fat between male broilers with high and low RFI.

**Conclusions:**

We identified QTL regions for BW28 and BW42 (spanning 0.32 Mb) and RFI (spanning 1.16 Mb). The *NSUN3*, *EPHA6*, and *AGK* were identified as the most likely candidate genes for these QTL. These genes are involved in mitochondrial function and behavioral regulation. These results contribute to the identification of candidate genes and variants for growth and feed efficiency in poultry.

## Background

Feed efficiency is the most important economic trait in poultry and livestock industries because up to 70% of the total production cost is due to feed [1]. Improvement of feed efficiency increases profitability for producers and reduces the environmental footprint of production. Feed efficiency is generally defined as the ability of an animal to convert feed nutrients into body mass or other useful products. The traditional measure of feed efficiency, feed conversion ratio (FCR), has been widely used in animal breeding programs. However, selection for FCR has not necessarily resulted in an improvement in the efficiency of feed utilization due to its strong correlation with body weight and growth rate [2]. An alternative measure of feed efficiency that has gained popularity in farm animal production is residual feed intake (RFI), which was first used by Koch et al. [3] for beef cattle. RFI is generally defined as the difference between the actual and the predicted feed intake during the measurement period. The predicted feed intake is based on the expected or average requirement of an animal for the maintenance of body weight and production [4] and is typically derived as the regression of ADFI on metabolic body weight and average daily gain (ADG). Compared with FCR, one of the main advantages of selection on RFI is reduction of feed intake without jeopardizing production traits, such as body weight and growth rate [5].

RFI-efficient animals tend to have leaner carcasses with lower subcutaneous fat thickness [6, 7]. Inclusion of backfat thickness in the model to account for difference in fat versus lean deposition when calculating RFI has been studied in cattle [8] and pigs [9]. In the current study, RFI adjusted for abdominal fat weight (RFIa) was calculated as the difference between the observed and the predicted ADFI using multiple regression of ADFI on metabolic body weight at mid-test (MWT), average daily gain (ADG), and abdominal fat (AbF) weight.

Quantitative trait loci (QTL) for traits in animals have been studied for more than 20 years. In chickens, the first QTL associated with FCR and RFI were detected on *Gallus gallus* (GGA) chromosome 4 by de Koning et al. [10]. Compared to the 594 QTL recorded for FCR across species, only 135 QTL for RFI are recorded in the Animal QTL Database (QTLdb; https://www.animalgenome.org/cgi-bin/QTLdb/GG/index, accessed 07 Jan 2021). Among these, five QTL were identified for RFI in chickens, on GGA3 (51.8–64.9 Mb), GGA10 (19.1–20.3 Mb), GGA12 (16.3–17.3 Mb), GGA19 (0.0–2.0 Mb), and GGAZ (2.1–7.8 Mb) [11, 12].

With the advent of high-density SNP genotyping arrays, genome-wide association studies (GWAS) have become a powerful tool for the detection of QTL in farm animals. GWAS for RFI have been performed in cattle [13–15], pigs [16], layers [17, 18], and meat-type chickens [12]. However, to date only a limited number of QTL for RFI have been identified in livestock. One reason is that RFI appears to be regulated by many genes, each with a small effect, and thus a relatively large sample size is required for the detection of these QTL [19].

Compared with cattle and pigs, generation of data on complex traits in a large population of chickens is relatively easy because of operational convenience, low unit cost of animals, short duration of the study, etc. The objectives of this study were to estimate genetic parameters, and identify significant loci and genes that affect feed efficiency traits in 3314 broilers, based on genotyping with a customized 55 K chicken single nucleotide polymorphism (SNP) array [20].

## Methods

### Experimental birds

The chickens used in this study were obtained from the pure line B of fast-growing chickens that has been selected for seven generations for increased body weight and growth rate traits by Foshan Gaoming Xinguang Agricultural and Animal Industrials Co., Ltd. (Foshan, China). The statistics of selection pressure in male and female selection candidates for generations 2 to 7 are in Additional file 1: Table S1. Body weight and feed intake were measured during the growth phase (28 to 41 days of age) on 2000 male and 1365 female chickens from generations 5 to 7 across 11 hatches. Among these, 1137 males and all females were selected according to hatch number and slaughtered at 42 days of age to obtain phenotypes on carcass traits, including abdominal fat (AbF) weight (see Additional file 2: Table S2). During the test period from 28 to 41 days of age, broilers were housed in individual cages with free access to feed and water. The corn-soybean meal diet contained 12.35 MJ/kg metabolic energy and 178 g/kg crude protein. Blood samples for DNA were obtained at 40 days of age via wing vein punctures using citrated syringes during a routine health inspection.

### Phenotypes

The eight traits that were either measured or calculated consisted of two growth traits, five feed intake and efficiency traits, and AbF weight. Growth traits were body weight at 28 days (d) of age (BW28) and BW42. Feed intake and efficiency traits were average daily feed intake (ADFI), residual feed intake (RFI), residual feed intake adjusted for weight of abdominal fat (RFIa), average daily gain (ADG), and feed conversion ratio (FCR). Total feed intake for each broiler for the total test period was used to calculate ADFI. Average daily gain (ADG) was calculated from BW28 and BW42. FCR was obtained from total feed intake divided by total weight gain from 28 to 42 days of age. Metabolic weight at mid-test (MWT) was calculated for each bird as the average of BW28 and BW42 (MBW) raised to the power of 0.75 (MBW^0.75^). After slaughter at 42 days of age, abdominal fat was removed and weighed for each individual to obtain AbF. RFI was estimated as the difference between the observed and the predicted ADFI with (RFIa) or without accounting for AbF. RFI and RFIa were derived as the residuals from the following two models:

$$\mathrm{ADFI}=\mu +\mathrm{hatch}+\mathrm{sex}+{\beta }_{1}\mathrm{MWT}+{\beta }_{2}\mathrm{ADG}+{e}_{1},$$

$$\mathrm{ADFI}=\mu +\mathrm{hatch}+\mathrm{sex}+{\beta }_{1}\mathrm{MW}T+{\beta }_{2}\mathrm{ADG}+{\beta }_{3}\mathrm{AbF}+{e}_{2},$$

where $\mu$ is the intercept, hatch and sex are fixed effects, MWT, ADG, and AbF are as defined above, ${\beta }_{1}$, ${\beta }_{2}$, and ${\beta }_{3}$ are partial regression coefficients, and $e$ is the residual.

Quality control was applied to the phenotypes of all traits and data on 35 birds with phenotypes that deviated by more than three standard deviations from the mean were removed.

### Genotyping, imputation, and quality control

Genomic DNA was extracted from blood samples using the phenol–chloroform method. Genotyping was conducted with a custom chicken 55 K SNP array (Beijing Compass Biotechnology Co., Ltd., Beijing, China), which is designed based on the Gallus_gallus-5.0 assembly and includes 52,060 SNPs [20]. The physical positions of these SNPs were liftovered to the GRCg6a assembly using the UCSC liftOver tool (20 May 2019). For quality control of the genotypic data, we used the PLINK (version 1.9) software [21]. Sixteen birds with a sample call rate lower than 90% were excluded. In total, 10,809 SNPs were removed with a call rate lower than 90% or a minor allelic frequency (MAF) lower than 0.05. Eighty-seven SNPs that were not assigned to chromosome or linkage group were also excluded. Missing alleles were imputed using the Beagle 5.0 software [22]. Finally, 41,164 SNPs and 1972 males and 1342 females passed the quality control criteria (see Additional file 3 Table S3).

### Estimation of heritability and genetic correlations

Univariate and bivariate animal models were fitted by restricted maximum likelihood (REML), using the ASReml v4.1 software package [23]. In preliminary analyses, the fixed effects of hatch and sex were significant (*P* < 0.01) based on Wald *F* statistics and were, therefore, included in the subsequent analyses. The following model with maternal genetic and maternal environmental effects was used:

$$\mathbf{y}=\mathbf{X}\mathbf{b}+{\mathbf{Z}}_{1}\mathbf{a}+{\mathbf{Z}}_{2}\mathbf{m}+{\mathbf{Z}}_{3}\mathbf{c}+\mathbf{e},$$

where $\mathbf{y}$ is the vector of observations, $\mathbf{b}$ is the vector of fixed effects, including hatch and sex, $\mathbf{a}$ is the vector of random direct additive genetic effects, $\mathbf{m}$ is the vector of random maternal additive genetic effects, $\mathbf{c}$ is the vector of random common maternal environmental effects, $\mathbf{e}$ is the vector of random residual effects, and $\mathbf{X}$, ${\mathbf{Z}}_{1}$, ${\mathbf{Z}}_{2}$, and ${\mathbf{Z}}_{3}$ are incidence matrices for $\mathbf{b}$, $\mathbf{a}$, $\mathbf{m}$, and $\mathbf{c}$, respectively. The variance–covariance structure assumed for random effects was:

$$\mathrm{Var}\left[\begin{array}{c}\mathbf{a}\\ \mathbf{m}\\ \mathbf{c}\\ \mathbf{e}\end{array}\right]=\left[\begin{array}{cccc}\mathbf{H}{\upsigma }_{\mathrm{a}}^{2}& \mathbf{H}{\upsigma }_{\mathrm{am}}& 0& 0\\ \mathbf{H}{\upsigma }_{\mathrm{am}}& \mathbf{H}{\upsigma }_{\mathrm{m}}^{2}& 0& 0\\ 0& 0& \mathbf{I}{\upsigma }_{\mathrm{c}}^{2}& 0\\ 0& 0& 0& \mathbf{I}{\upsigma }_{\mathrm{e}}^{2}\end{array}\right],$$

where ${\upsigma }_{\mathrm{a}}^{2}$, ${\upsigma }_{\mathrm{m}}^{2}$, ${\upsigma }_{\mathrm{c}}^{2}$, and ${\upsigma }_{\mathrm{e}}^{2}$ are the direct additive genetic, maternal additive genetic, common maternal environmental, and residual error variances, respectively, ${\upsigma }_{\mathrm{am}}$ is the covariance between direct and maternal genetic effects, $\mathbf{H}$ is a relationship matrix that combines genomic and pedigree relationships because 1304 dams (including 675 dams from generation 4) had only pedigree information, and $\mathbf{I}$ is an identity matrix. The covariance between direct and maternal genetic effects was assumed to be zero when convergence problems were encountered. Matrix $\mathbf{H}$ was constructed as in Legarra et al. [24]:

$$\mathbf{H}=\left[\begin{array}{cc}{\mathbf{A}}_{11}+{\mathbf{A}}_{12}{\mathbf{A}}_{22}^{-1}(\mathbf{G}-{\mathbf{A}}_{22}){\mathbf{A}}_{22}^{-1}{\mathbf{A}}_{21}& {\mathbf{A}}_{12}{\mathbf{A}}_{22}^{-1}\mathbf{G}\\ \mathbf{G}{\mathbf{A}}_{22}^{-1}{\mathbf{A}}_{21}& \mathbf{G}\end{array}\right],$$

where ${\mathbf{A}}_{11}$, ${\mathbf{A}}_{12}$, ${\mathbf{A}}_{21}$, and ${\mathbf{A}}_{22}$ are submatrices extracted from the pedigree-based relationship matrix $\mathbf{A}$**,** with indices 1 for non-genotyped dams and 2 for genotyped animals, and $\mathbf{G}$ is the genomic relationship matrix based on the SNPs following the first method of VanRaden [25]. The same model was also used for pedigree-based analyses by replacing $\mathbf{H}$ by the pedigree-based relationship matrix $\mathbf{A}$. The pedigree used in the analysis consisted of 4865 birds from generations 3 to 7.

### Genome-wide association study

A GWAS was performed for each trait using a univariate linear mixed model implemented in GEMMA version 0.98.1 software (https://github.com/genetics-statistics/GEMMA/releases) [26], one SNP at a time. The fixed effects of hatch and sex were included in the model for all traits, except for RFI and RFIa. The hatch and sex were included as fixed effects when the phenotypic values of RFI and RFIa were estimated. The fixed effects were used to construct a design matrix using the model.matrix() function in R (version 3.6.0) and included in the GEMMA software similar to a covariate. The statistical model used was:

$$\mathbf{y}=\mathbf{X}\mathbf{b}+\mathbf{S}\beta +\mathbf{u}+\mathbf{e},$$

$$\mathbf{u}\sim \mathrm{MVN}\left(0, \mathbf{G}{\upsigma }_{\mathrm{a}}^{2}\right),$$

$$\mathbf{e}\sim \mathrm{MVN}\left(0,{\mathbf{I}\upsigma }_{\mathrm{e}}^{2}\right),$$

where $\mathbf{y}$ is a vector of phenotypic values, $\mathbf{X}$ is the design matrix for fixed effects, including a column of 1 s, $\mathbf{b}$ is the vector of the corresponding effect estimates, including the intercept, $\mathbf{S}$ is a vector of genotypes coded major/minor alleles as 0/1 for a given SNP, $\beta$ is the allele substitution effect for the fitted SNP, $\mathbf{u}$ is a vector of random animal genetic effects, $\mathbf{e}$ is a vector of residuals, ${\upsigma }_{\mathrm{a}}^{2}$ is the direct additive genetic variance, ${\upsigma }_{\mathrm{e}}^{2}$ is the residual error variance, $\mathbf{G}$ is the genomic relationship matrix, $\mathbf{I}$ is an identity matrix, and $\mathrm{MVN}$ is the multivariate normal distribution.

The Wald test was used as a criterion to identify SNPs that were significantly associated with the investigated traits. Genome-wide significance was assessed using the simple*M* method [27]. This method first derives the composite linkage disequilibrium (LD) correlation matrix from the SNP genotypes, then calculates the eigenvalues of the component LD matrix by principal component analysis, and finally infers the effective number of independent tests as the number of principal components that jointly contribute 99.5% of the variation in SNP genotypes. Manhattan and quantile–quantile (Q-Q) plots were constructed for each trait by the qqman package (http://cran.r-project.org/package=qqman) in R (version 3.6.0). The genomic inflation factor (GIF) was calculated by the GenABEL R package [28]. The percentage of genetic variance that was explained by each significant SNP was calculated following Al-Mamun et al. [29] as: $\mathrm{\%}{V}_{gi}=\frac{2{p}_{i}{q}_{i}{\beta }_{i}^{2}}{{\sigma }_{g}^{2}}\times 100$, where ${p}_{i}$ and ${q}_{i}$ are the allele frequencies for SNP $i$, ${\beta }_{i}$ is the estimate of the allele substitution effect of SNP $i$ based on the GWAS model, and ${\sigma }_{g}^{2}$ is the estimate of genetic variance for the trait estimated as described above using the combined genomic-pedigree relationship matrix $\mathbf{H}$.

### Further analysis of QTL regions

Significant and adjacent SNPs for the analyzed traits were further investigated by region-based association analyses using the fast family-based sequence kernel association test (FFBSKAT) program (SNP effects fitted as random effects) implemented in the R package FREGAT [30]. Region-based association analysis is an efficient approach to identify causal SNPs. The fixed effects of hatch and sex were added to the FFBSKAT, except for RFI and RFIa. The linear mixed model was given following Schifano et al. [31]: $\mathbf{y}=\mathbf{X}\mathbf{b}+\mathbf{h}+\mathbf{u}+\mathbf{e}$, where $\mathbf{h}$ is the vector of random SNP effects and the other parameters are the same as for the GEMMA model. The LD among SNPs in these regions was estimated using PLINK (version 1.9) [21]. Boxplots of the phenotype distribution by genotype of SNPs were produced by the ggplot2 package in R (version 3.6.0).

Additive and dominance effects of SNPs on the analyzed traits were estimated using the ASReml v4.1 software package [23]. To estimate the additive effect, a covariate was fitted with values 1, 0, and -1 for the homozygous genotype for the major allele, the heterozygous genotype, and the homozygous genotype for the minor allele, respectively. To estimate the dominance effect, a covariate was fitted with value 0 for the homozygous genotypes and 1 for the heterozygous genotype. The model was: $\mathbf{y}=\mathbf{X}\mathbf{b}+\mathbf{Q}\mathbf{q}+\mathbf{u}+\mathbf{e}$, where $\mathbf{Q}$ is the design matrix for a SNP (additive and dominance) effects, $\mathbf{q}$ is the additive and dominance effects for the fitted SNP, other components are as defined for the GEMMA model.

Allele frequencies at the most significant SNPs in three generations (generations 5, 6, and 7) were estimated using PLINK (version 1.9) [21].

### Analysis of the expression of candidate genes by qPCR

The genes that were located closest to genome-wide significant and suggestive SNPs were identified based on the UCSC annotation of the GRCg6a genome version (http://genome-asia.ucsc.edu/cgi-bin/hgGateway?hgsid=472768848_otkBtCHKhHMTV1xrxHuq737iivJ1). Expression of these candidate genes (*NSUN3*, *EPHA6*, and *AGK*) was assessed in the relevant tissues using qPCR analyses. One hundred and seventy-five males from generation 6 with growth and feed efficiency between 28 and 41 d of age were slaughtered at 42 d of age to measure carcass traits. Tissue samples from birds with the highest (n = 15) and lowest (n = 15) RFI phenotypes were collected (Table 1).

**Table 1:** Means (± SD) for growth, feed efficiency, and carcass traits for the high and low RFI cohorts^a^

Traits^b^	High RFI	Low RFI
BW28 (g)	1231 ± 81	1195 ± 82
BW42 (g)	2448 ± 166	2428 ± 134
ADFI (g/d)	176.3 ± 11.3^**^	156.3 ± 8.4
ADG (g/d)	86.95 ± 9.57	88.07 ± 5.86
RFI (g/d)	9.39 ± 3.48^**^	− 10.71 ± 2.06
RFIa (g/d)	9.99 ± 2.86^**^	− 7.13 ± 2.81
FCR (g/g)	2.04 ± 0.12^**^	1.78 ± 0.05
CW (g)	2184 ± 156	2164 ± 109
BrW (g)	518.0 ± 42.1	546.1 ± 49.5
ThW (g)	395.7 ± 48.0	394.4 ± 31.3
AbF (g)	31.61 ± 10.24^**^	20.48 ± 8.65
CWP (%)	89.18 ± 0.84	89.67 ± 2.83
BrP (%)	21.17 ± 1.06	22.47 ± 1.29^**^
ThP (%)	16.12 ± 1.11	16.23 ± 0.52
AbP (%)	1.30 ± 0.42^**^	0.84 ± 0.35

^a^High RFI group (n = 15) consisting of samples from generation 6 of male broilers with the highest RFI values and low RFI group (n = 15) with the lowest RFI values; **P < 0.01

^b^BW28, body weight at 28 d of age; BW42, body weight at 42 d of age; ADFI, average daily feed intake; ADG, average daily gain; RFI, residual feed intake; RFIa, residual feed intake adjusted for weight of abdominal fat; FCR, feed conversion ratio; CW, carcass weight; BrW, breast muscle weight; ThW, thigh muscle weight; AbF, weight of abdominal fat; CWP, percentage of carcass weight; BrP, percentage of breast muscle; ThP, percentage of thigh muscle; AbP, percentage of abdominal fat

Total RNA was isolated from liver, breast muscle, thigh muscle, and AbF tissues using the TRIzol reagent (Life Technologies, Inc., Carlsbad, CA). First-strand cDNA was synthesized from 2 µg total RNA using the FastQuant RT Kit (with gDNAase) (TIANGEN BIOTECH, Beijing, China). Power SYBR® Fast qPCR Master Mix (KAPA, Wilmington, MA) was used to analyze mRNA expression of the selected genes. qPCR analysis was performed with an ABI Q7 Flex Real-time detection system (Applied Biosystems, Foster City, CA). Specific primers [see Additional file 4 Table S4] were designed using the Primer Premier 6.0 software based on chicken coding sequences and were subsequently synthesized by the Beijing Genomics Institute (Beijing, China). The desired PCR product size was set to 100 to 250 bp and the best primer pair among the output was selected.

Amplification was performed in a total volume of 10 µL, containing 5 µL of 2 × PCR Master Mix, 0.2 µL of 50 $\times$ ROX Low Reference Dye, 1.2 µL cDNA (100 ng/µL), 0.3 µLl of each primer, and 3.0 µL ddH_2_O. The PCR cycle parameters were: 95 °C for 3 min, then 40 amplification cycles at 95 °C for 3 s and 60 °C for 34 s. The following housekeeping genes were used as reference genes: *ribosomal protein L32* (*RPL32*) for breast muscle and thigh muscle [32], *ubiquitin B* (*UBB*) for liver tissue [33], and *glyceraldehyde-3-phosphate dehydrogenase* (*GAPDH*) for AbF tissue [34]. The comparative CT method [35] was used to determine fold-changes in gene expression calculated as ${2}^{-\mathrm{\Delta \Delta CT}}$. Statistical analyses were performed using analysis of variance (general linear model) and Tukey’s test in SAS 9.4 (SAS Institute, Cary, NC) [36]. Threshold significance values were *P* < 0.05 (*) or *P* < 0.01 (**).

## Results

### Descriptive statistics of growth and feed efficiency traits

The descriptive statistics in Table 2 show the distribution of growth and feed efficiency traits of the 3314 broilers (1972 males and 1342 females) used in the GWAS. Phenotypes for individual broilers [see Additional file 5: Table S5] and descriptive statistics for male and female broilers are shown separately [see Additional file 6: Table S6]. The average body weight at 28 days of age (BW28) was 1.08 kg and reached 2.21 kg at 42 days of age (BW42). Chickens consumed an average of 151 g/days of feed and gained 80.6 g/days in weight during the growth phase (28 to 41 days of age). Values of RFI and RFIa ranged from -19.3 to 19.0 g/days and from -18.0 to 18.1 g/days, respectively. The coefficients of variation for the five growth and feed efficiency traits ranged from 7.8 to 19.5%.

**Table 2:** Descriptive statistics for the evaluated growth and feed efficiency traits

Traits	N	Mean	SD	Min	Max	CV (%)^b^
BW28 (g)	3314	1078	176	557	1530	16.32
BW42 (g)	3314	2206	365	1198	3120	16.55
ADFI (g/d)	3314	151.3	25.4	88.2	222.0	16.82
RFI (g/d)	3314	0.00	6.00	− 19.33	19.03	–
RFIa (g/d)	2453	0.00	5.84	− 17.99	18.13	–
ADG (g/d)	3314	80.56	15.72	40.43	127.86	19.52
FCR (g:g)	3314	1.89	0.15	1.43	2.39	7.77
AbF (g)	2453	34.73	11.17	1.20	90.80	32.17

BW28, body weight at 28 d of age; BW42, body weight at 42 d of age; ADFI, average daily feed intake; RFI, residual feed intake; RFIa, residual feed intake adjusted for weight of abdominal fat; ADG, average daily gain; FCR, feed conversion ratio; AbF, weight of abdominal fat; CV, coefficient of variation

### Genetic parameters of growth and feed efficiency traits

Estimates of variance components and heritabilities using genomic and pedigree relationship matrices are in Table 3. Estimates of genomic heritabilities for two growth traits (BW28 and BW42) and the five feed efficiency traits (ADFI, RFI, RFIa, ADG, and FCR) were low to moderate, ranging from 0.12 to 0.26. Genetic correlations between direct and maternal effects for BW28, BW42, ADFI, RFI, RFIa, and AbF were moderate to high, ranging from -0.77 to -0.45. Estimates of heritabilities and genetic correlations between direct and maternal effects based on pedigree information were higher than genomic estimates. Estimates of maternal genetic and maternal environmental effects on growth and feed efficiency traits were low (0.00 to 0.09). Estimates of variance components and heritabilities based on genomic and pedigree relationship matrices are shown separately for male and female broilers in Additional file 7: Table S7 and Additional file 8: Table S8. Estimates of additive genetic variances and heritabilities of growth and feed efficiency traits were lower for males than for females.

**Table 3:** Estimates of variance components and heritabilities for growth and feed efficiency traits using genomic and pedigree relationship matrices

Traits	Matrix^b^	Parameters^c^								
		${\upsigma }_{\mathrm{a}}^{2}$	${\upsigma }_{\mathrm{m}}^{2}$	${\sigma }_{\mathrm{am}}$	${\upsigma }_{\mathrm{c}}^{2}$	${\upsigma }_{\mathrm{e}}^{2}$	${\mathrm{h}}_{\mathrm{a}}^{2}$	${\mathrm{h}}_{\mathrm{m}}^{2}$	${\mathrm{r}}_{\mathrm{am}}$	c^2^
BW28	H	1904 ± 363	842.8 ± 432.7	− 566.8 ± 361.8	190.6 ± 292.4	7125 ± 316	0.20 ± 0.04	0.09 ± 0.05	− 0.45 ± 0.20	0.02 ± 0.03
	A	3828 ± 756	707.6 ± 473.4	− 1642 ± 546	486.2 ± 286.8	6368 ± 463	0.39 ± 0.07	0.07 ± 0.05	− 1.00 ± 0.16	0.05 ± 0.03
BW42	H	5735 ± 959	1845 ± 983	− 2513 ± 909	658.3 ± 641.1	16,114 ± 761	0.26 ± 0.04	0.08 ± 0.04	− 0.77 ± 0.15	0.03 ± 0.03
	A	5951 ± 1,165	–	–	37.53 ± 510.75	16,358 ± 867	0.27 ± 0.05	–	–	0.00 ± 0.02
ADFI	H	30.13 ± 5.14	10.56 ± 4.94	− 12.86 ± 4.66	1.49 ± 3.29	89.05 ± 4.18	0.25 ± 0.04	0.09 ± 0.04	− 0.72 ± 0.14	0.01 ± 0.03
	A	41.38 ± 8.66	9.42 ± 5.46	− 16.86 ± 6.07	0.61 ± 3.34	86.30 ± 5.55	0.34 ± 0.07	0.08 ± 0.04	− 0.85 ± 0.13	0.01 ± 0.03
RFI	H	8.89 ± 1.52	2.42 ± 1.33	− 2.98 ± 1.31	–	27.20 ± 1.26	0.25 ± 0.04	0.07 ± 0.04	− 0.64 ± 0.17	–
	A	9.73 ± 2.25	1.25 ± 1.39	− 3.15 ± 1.56	–	28.25 ± 1.56	0.27 ± 0.06	0.03 ± 0.04	− 0.90 ± 0.27	–
RFIa	H	6.05 ± 1.55	1.10 ± 1.66	− 1.52 ± 1.49	0.51 ± 1.24	27.52 ± 1.49	0.18 ± 0.05	0.03 ± 0.05	− 0.59 ± 0.39	0.02 ± 0.04
	A	6.53 ± 2.27	1.96 ± 2.09	− 3.16 ± 1.95	0.36 ± 1.29	28.47 ± 1.71	0.19 ± 0.06	0.06 ± 0.06	− 0.88 ± 0.25	0.01 ± 0.04
ADG	H	6.06 ± 1.25	–	–	1.73 ± 0.98	39.58 ± 1.55	0.13 ± 0.03	–	–	0.04 ± 0.02
	A	6.84 ± 1.77	–	–	0.69 ± 1.07	40.04 ± 1.68	0.14 ± 0.04	–	–	0.01 ± 0.02
FCR	H	0.0015 ± 0.0003	–	–	0.0001 ± 0.0002	0.0104 ± 0.0004	0.12 ± 0.02	–	–	0.01 ± 0.02
	A	0.0015 ± 0.0004	–	–	–	0.0105 ± 0.0004	0.12 ± 0.03	–	–	–
AbF	H	42.48 ± 5.89	18.47 ± 6.23	− 17.92 ± 5.67	2.01 ± 3.65	48.83 ± 3.81	0.45 ± 0.06	0.20 ± 0.06	− 0.64 ± 0.12	0.02 ± 0.04
	A	59.36 ± 11.93	17.58 ± 7.68	− 24.94 ± 8.11	0.78 ± 3.95	46.00 ± 6.72	0.60 ± 0.11	0.18 ± 0.08	− 0.77 ± 0.10	0.01 ± 0.04

BW28, body weight at 28 d of age; BW42, body weight at 42 d of age; ADFI, average daily feed intake; RFI, residual feed intake; RFIa, residual feed intake adjusted for weight of abdominal fat; ADG, average daily gain; FCR, feed conversion ratio; AbF, weight of abdominal fat

^b^H, the relationship matrix that blends genomic and pedigree information; A, the pedigree-based relationship matrix

^c^ ${\upsigma }_{\mathrm{a}}^{2}$, direct additive genetic variance; ${\upsigma }_{\mathrm{m}}^{2}$, maternal additive genetic variance; ${\sigma }_{\mathrm{am}}$, covariance between direct and maternal genetic effects; ${\upsigma }_{\mathrm{c}}^{2}$, common maternal environment variance;${\upsigma }_{\mathrm{e}}^{2}$, residual error variance; ${\mathrm{h}}_{\mathrm{a}}^{2}$, direct heritability; ${\mathrm{h}}_{\mathrm{m}}^{2}$, maternal heritability; ${\mathrm{r}}_{\mathrm{am}}$, genetic correlation between direct and maternal effects; c^2^, maternal environmental variance as a proportion of phenotypic variance. — represents close to zero

Estimates of genetic (${\mathrm{r}}_{\mathrm{g}}$) and phenotypic (${\mathrm{r}}_{\mathrm{p}}$) correlations using the combined genomic-pedigree relationship matrix are in Table 4. Estimates based on the pedigree relationship matrix were very similar ($\mathrm{r}$ = 0.97, [see Additional file 9 Table S9]). Estimates of genetic correlations of ADFI with BW28, BW42, RFI, RFIa, ADG, and AbF were moderate to high, ranging from 0.45 to 0.83, and was lowest (0.26) with FCR (Table 4). Estimates of genetic correlations of BW28 and BW42 with RFI, RFIa, and FCR ranged from -0.09 to 0.15 and did not significantly differ from zero. Estimates of phenotypic correlations of ADG with RFI and RFIa were zero, and the genetic correlations were low (0.17 with RFI and 0.19 with RFIa). Estimates of genetic correlations of AbF with RFI and FCR were 0.48 and 0.37, respectively, whereas that of AbF with RFIa was 0.05. Estimates of genetic and phenotypic correlations of RFI with RFIa and FCR were high and positive (r > 0.71).

**Table 4:** Estimates of genetic and phenotypic correlations between growth and feed efficiency traits using the relationship matrix that blends genomic and pedigree information

Traits^a^	BW28	BW42	ADFI	RFI	RFIa	ADG	FCR	AbF
BW28		0.87 ± 0.03	0.50 ± 0.08	− 0.09 ± 0.10	− 0.02 ± 0.12	0.39 ± 0.10	0.15 ± 0.12	0.23 ± 0.09
BW42	0.77 ± 0.01		0.77 ± 0.04	0.03 ± 0.10	0.09 ± 0.12	0.79 ± 0.04	− 0.08 ± 0.12	0.26 ± 0.08
ADFI	0.41 ± 0.02	0.78 ± 0.01		0.63 ± 0.07	0.57 ± 0.09	0.83 ± 0.04	0.26 ± 0.12	0.45 ± 0.08
RFI	− 0.02 ± 0.02	− 0.01 ± 0.02	0.55 ± 0.01		0.83 ± 0.03	0.17 ± 0.12	0.75 ± 0.06	0.48 ± 0.08
RFIa	− 0.01 ± 0.02	− 0.01 ± 0.02	0.47 ± 0.02	0.92 ± 0.00		0.19 ± 0.14	0.57 ± 0.09	0.05 ± 0.11
ADG	0.17 ± 0.02	0.76 ± 0.01	0.79 ± 0.01	0.00 ± 0.02	0.00 ± 0.02		− 0.33 ± 0.12	0.19 ± 0.10
FCR	0.26 ± 0.02	− 0.19 ± 0.02	0.05 ± 0.02	0.71 ± 0.01	0.61 ± 0.01	− 0.56 ± 0.01		0.37 ± 0.10
AbF	0.29 ± 0.02	0.35 ± 0.02	0.46 ± 0.02	0.32 ± 0.02	− 0.01 ± 0.02	0.26 ± 0.02	0.17 ± 0.02	

^a^Upper diagonal is genetic correlation, and lower diagonal is phenotypic correlation

BW28, body weight at 28 d of age; BW42, body weight at 42 d of age; ADFI, average daily feed intake; RFI, residual feed intake; RFIa, residual feed intake adjusted for weight of abdominal fat; ADG, average daily gain; FCR, feed conversion ratio; AbF, weight of abdominal fat

### Genome-wide association study of growth and feed efficiency traits

The number of independent tests was estimated to be 26,217. Based on this, genome-wide and suggestive significance thresholds were set equal to 1.91*10^–6^ (0.05/26,217) and 3.81*10^–5^ (1/26,217), respectively.

#### Growth traits

**Fig. 1:**
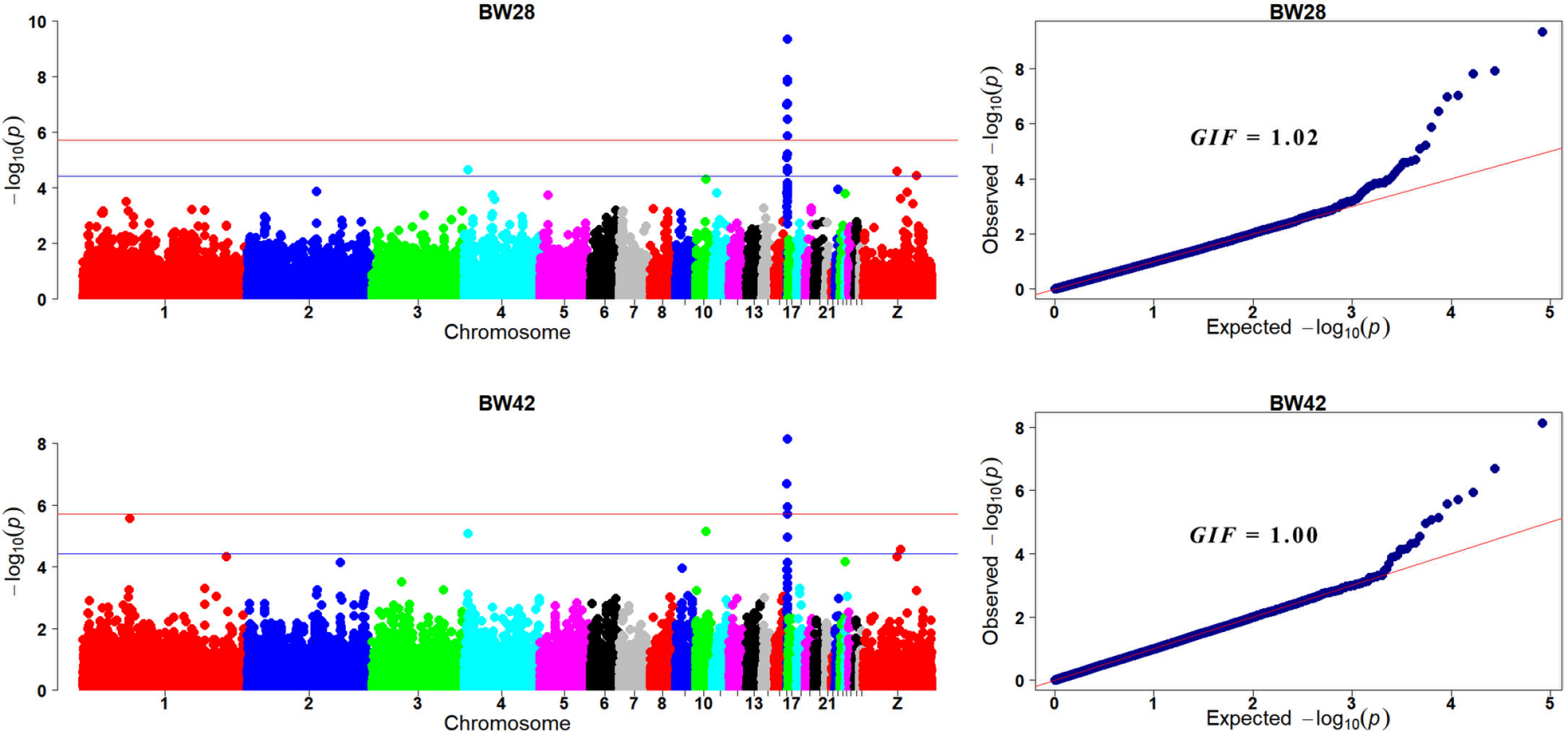
Manhattan and quantile–quantile (Q-Q) plots of the GWAS for growth traits. Each dot is a SNP in the dataset. The horizontal red and blue lines indicate the genome-wide significant (*P* value = 1.90e−6) and suggestive thresholds (*P* value = 3.80e−5), respectively. BW28 and BW42 are body weight at 28 d and 42 d of age, and GIF is the genomic inflation factor

Manhattan and quantile–quantile (Q-Q) plots for BW28 and BW42 are shown in Fig. 1. Detailed information on SNPs significantly associated with BW28 and BW42 is in Table 5 and sequence information regarding these SNPs is in Additional file 10: Table S10.

**Table 5:** Information on SNPs associated with growth traits

Traits^a^	SNPs	rsname^b^	GGA^c^	Position	Alleles	MAF	*β*^d^	Var (%)^e^	*P* value	Candidate gene	Distance (kb)^f^
BW28	AX_76670473	rs14422146	4	4,486,368	C/T	0.15	16.35	3.56	2.23e−05	*TM9SF2L*	D 33.97
BW28	AX_172583411	rs732597861	16	2,342,047	G/T	0.34	− 11.15	2.92	1.02e−07	*BG8*	U 47.43
BW28	AX_172583423	rs794119717	16	2,399,483	T/G	0.39	9.75	2.37	8.07e−06	*BG8*	D 3.59
BW28	AX_172583407	NA	16	2,451,685	C/T	0.26	− 16.75	5.61	9.08e−08	*ZNF692*	Intron 8
BW28	AX_172583410	rs736020965	16	2,455,748	A/G	0.44	− 11.83	3.62	1.32e−06	*TRIM7.2*	Intron 6
BW28	AX_172583409	rs739811469	16	2,469,604	G/A	0.27	13.63	3.87	1.94e−05	*BZFP1*	D 2.03
BW28	AX_101003762	rs15788030	16	2,502,939	T/C	0.26	− 19.26	7.56	4.41e−10	*TRIM39.2*	Intron 2
BW28	AX_75851417	rs312648889	16	2,506,461	T/C	0.41	13.32	4.50	5.81e−06	*TRIM27.2*	Exon 1
BW28	AX_75851232	rs15788124	16	2,537,500	C/T	0.12	− 16.62	3.11	2.52e−05	*RACK1*	D 7.68
BW28	AX_75851146	rs316225723	16	2,548,043	T/C	0.16	− 21.62	6.50	1.21e−08	*BG1*	U 2.17
BW28	AX_75852151	rs16042753	16	2,630,726	G/A	0.20	20.24	6.83	1.52e−08	*CYP21A1*	D 0.03
BW28	AX_75851193	rs315640666	16	2,658,014	G/A	0.41	− 14.44	5.32	3.42e−07	*CD1B*	U 4.15
BW42	AX_172570918	rs314078158	1	5,6289,810	T/C	0.17	− 25.08	3.11	2.70e−06	*ATP6V0A4*	U 39.37
BW42	AX_76670473	rs14422146	4	4,486,368	C/T	0.15	25.83	2.95	8.29e−06	*TM9SF2L*	D 33.97
BW42	AX_172581004	rs731588889	10	13,578,799	C/T	0.11	− 29.08	2.95	7.14e−06	*MRPS11*	U 33.53
BW42	AX_172583411	rs732597861	16	2,342,047	G/T	0.34	− 16.40	2.10	1.98e−07	*BG8*	U 47.43
BW42	AX_172583424	rs741681687	16	2,419,674	A/C	0.40	21.15	3.75	1.16e−06	*KIFC1*	U 19.46
BW42	AX_172583407	NA	16	2,451,685	C/T	0.26	− 27.29	4.95	7.17e−09	*ZNF692*	Intron 8
BW42	AX_101003762	rs15788030	16	2,502,939	T/C	0.26	− 22.19	3.33	1.94e−06	*TRIM39.2*	Intron 2
BW42	AX_75851146	rs316225723	16	2,548,043	T/C	0.16	− 25.15	2.92	1.10e−05	*BG1*	U 2.17

^a^BW28, body weight at 28 d of age; BW42, body weight at 42 d of age

^b^NA: not available

^c^*Gallus gallus* chromosome

^d^Allele substitution effect was the additive effect estimated by GEMMA

^e^The proportion of the genetic variance accounted by the SNP

^f^U and D indicate that the SNP is upstream and downstream of a gene, respectively

For BW28, seven genome-wide significant SNPs were identified on GGA16, and five suggestively significant SNPs were detected on GGA4 and 16. The genomic inflation factor (GIF) was 1.02 for BW28, which suggests that the population structure was well controlled. Eleven of the genome-wide significant SNPs were within a 316 kb region on GGA16 (2.34–2.66 Mb). The minor allele of the most significant SNP in this region, AX_101003762, had a negative effect estimate ($\beta <0$) and accounted for 7.6% of the genetic variance for BW28. These SNPs on GGA16 were located either within genes or 0.03 to 47.43 kb away from the nearest genes, which include *zinc finger protein 692* (*ZNF692*), *tripartite motif containing 39.2* (*TRIM39.2*), and *tripartite motif containing 27.2* (*TRIM27.2*).

For BW42, three genome-wide significant SNPs and five suggestively significant SNPs were located on GGA1, 4, 10, and 16. The GIF was 1.00, which indicates that this association analysis was hardly affected, if at all, by population stratification. Three genome-wide significant SNPs and two suggestively significant SNPs were located within a 206.0 kb region (GGA16: 2.34–2.55 Mb) and four SNPs were the same as those found for BW28. The leading SNP for BW42, AX_172583407, accounted for 5.0% of the genetic variance.

#### Feed efficiency traits

**Fig. 2:**
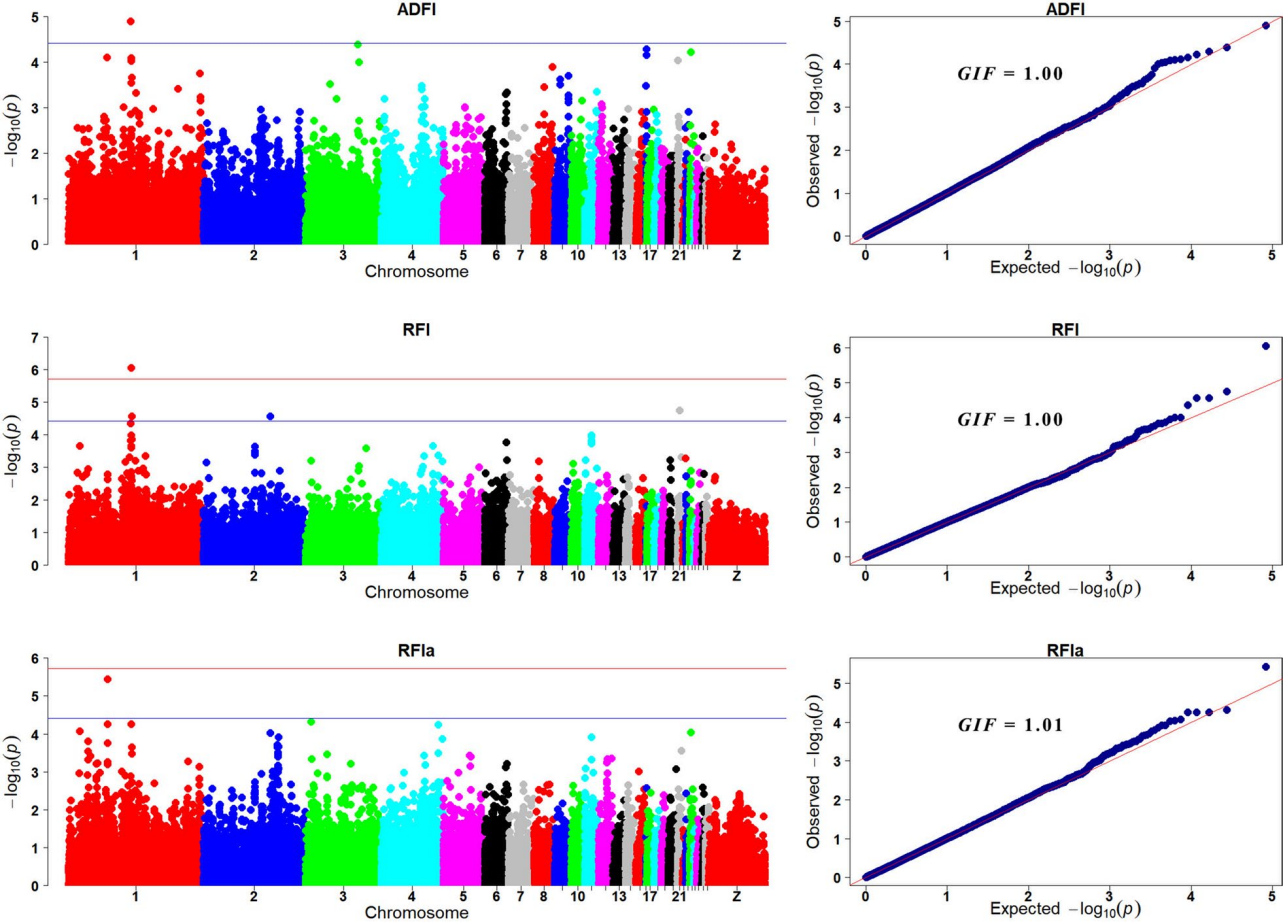
Manhattan and quantile–quantile (Q-Q) plots of the GWAS for feed efficiency traits. Each dot is a SNP in the dataset. The horizontal red and blue lines indicate the genome-wide significant (*P* value = 1.90e−6) and suggestive thresholds (*P* value = 3.80e−5), respectively. ADFI, RFI, and RFIa are average daily feed intake, residual feed intake, and residual feed intake adjusted for weight of abdominal fat, respectively, and GIF is the genomic inflation factor

Manhattan and quantile–quantile (Q-Q) plots for the three feed efficiency traits are shown in Fig. 2. Detailed information on SNPs significantly associated with the feed efficiency traits is in Table 6 and sequence information regarding these SNPs is in Additional file 10: Table S10.

**Table 6:** Information on SNPs associated with feed efficiency traits

Traits^a^	SNPs	rsname	GGA^b^	Position	Alleles	MAF	${\beta }^{c}$	Var (%)^d^	*P* value	Candidate gene	Distance (kb)^e^
ADFI	AX_75546765	rs13649171	1	91,274,115	C/A	0.15	− 2.02	3.37	1.24e−05	*NSUN3*	D 72.07
RFI	AX_172588157	rs740268684	1	91,974,671	C/T	0.38	− 0.91	4.44	8.62e−07	*EPHA6*	U 166.15
RFI	AX_172673005	rs312607889	1	92,432,544	C/T	0.27	− 0.86	3.22	2.74e−05	*EPHA6*	Intron 5
RFI	AX_80894722	rs314437326	2	98,063,070	C/G	0.29	0.79	2.91	2.72e−05	*NAPG*	Intron 2
RFI	AX_76242939	rs313748618	21	3,587,117	A/G	0.08	1.18	2.37	1.79e−05	*LZIC*	Intron 1
RFIa	AX_172566874	rs316086126	1	57,280,149	A/G	0.44	− 0.88	6.34	3.66e−06	*AGK*	Intron 1

^a^ADFI, average daily feed intake; RFI, residual feed intake; RFIa, residual feed intake adjusted for weight of abdominal fat

^b^*Gallus gallus* chromosome

^c^Allele substitution effect was the additive effect estimated by GEMMA

^d^The proportion of the genetic variance accounted by the SNP

^e^U and D indicate that the SNP is upstream and downstream of a gene, respectively

For ADFI, one SNP exceeded the suggestive significance threshold (GGA1: SNP AX_75546765 at 91.27 Mb). This SNP was located near the *NOP2/Sun RNA methyltransferase family member 3* (*NSUN3*) gene and accounted for 3.4% of the genetic variance for ADFI.

For RFI, one genome-wide significant SNP and three suggestively significant SNPs were detected, two of which were located within a 457.9 kb region on GGA1 (91.97–92.43 Mb). This region was located about 700.6 kb downstream of the SNP AX_75546765 that was associated with ADFI. These SNPs were located within or near the *EPH receptor A6* (*EPHA6*), *NSF attachment protein gamma* (*NAPG*), and *leucine zipper and CTNNBIP1 domain containing* (*LZIC*) genes. The leading variant, AX_172588157, accounted for 4.4% of the genetic variance for RFI.

For RFIa, one genome-wide suggestively significant SNP (GGA1: 57.28 Mb) was located in the first intron of the *acylglycerol kinase* (*AGK*) gene. This SNP accounted for 6.3% of the genetic variance.

### Region-based association test, linkage disequilibrium, and allele frequency analysis

**Fig. 3:**
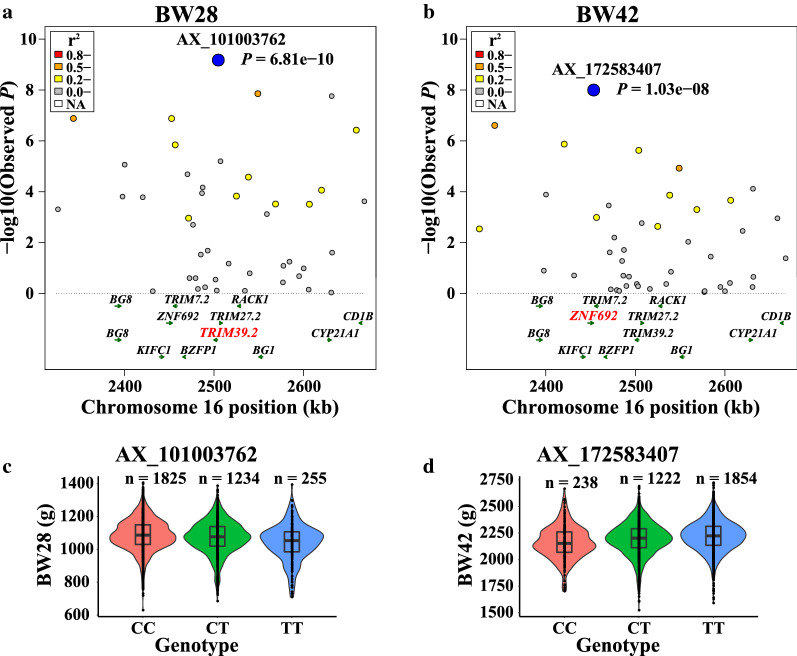
Association results for the candidate region on GGA16 for BW28 and BW42. **a** Regional plot of the candidate region on GGA16 (2.34–2.66 Mb) for BW28. **b** Regional plot of the candidate region on GGA16 (2.34–2.66 Mb) for BW42. In the upper panels, the leading SNPs are highlighted by blue solid circles and those near or within the gene in red color. Different levels of linkage disequilibrium between the leading SNP and surrounding SNPs are indicated in different colors. *P*-values are based on analyses in FFBSKAT. **c** Boxplot for BW28 and the genotype at SNP AX_101003762. **d** Boxplot for BW42 and the genotype at SNP AX_172583407. BW28 and BW42 are body weight at 28 d and 42 days of age

**Fig. 4:**
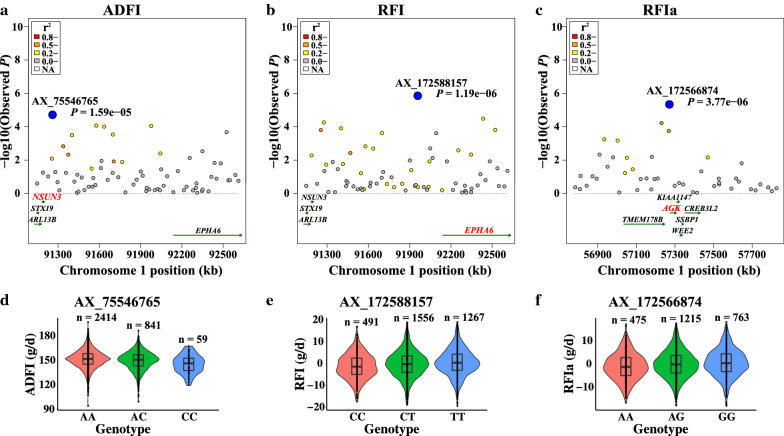
Association results for the candidate region on GGA1 for ADFI, RFI, and RFIa. **a** Regional plot of the candidate region on GGA1 (91.15–92.61 Mb) for ADFI. **b** Regional plot of the candidate region on GGA1 (91.15–92.61 Mb) for RFI. **c** Regional plot of the candidate region on GGA1 (56.78–57.82 Mb) for RFIa. In the upper panels, the leading SNPs are highlighted by blue solid circles and those near or within the gene by red color. Different levels of linkage disequilibrium between the leading SNP and surrounding SNPs are indicated in different colors. *P*-values are based on analyses in FFBSKAT. **d** Boxplot for ADFI and genotype at SNP AX_75546765. **e** Boxplot for RFI and genotype at SNP AX_172588157. **f** Boxplot for RFIa and genotype at SNP AX_172566874. ADFI, RFI, and RFIa are average daily feed intake, residual feed intake, and residual feed intake adjusted for weight of abdominal fat, respectively

The two region-based association plots for GGA16 and GGA1, which included multiple genes in the associated LD region, are shown in Figs. 3a–b and 4a–c. For one region on GGA16 (2.34–2.66 Mb), the strongest associated SNPs were AX_101003762 for BW28, within the *TRIM39.2* gene (*P* = 6.81e−10), and AX_172583407 for BW42, within the *ZNF692* gene (*P* = 1.03e−8). LD analysis showed that the levels of LD were moderate for several haplotype blocks. For two regions on GGA1 (91.15–92.61 Mb and 56.78–57.82 Mb), AX_75546765, AX_172588157, and AX_172566874 were identified as leading SNPs in the vicinity of the genes *NSUN3*, *EPHA6*, and *AGK*, for ADFI, RFI, and RFIa, respectively. The two regions also included other genes, including *TRIM27.2*, *receptor for activated C kinase 1* (*RACK1*), *kinesin family member C1* (*KIFC1*), *ADP ribosylation factor like GTPase 13B* (*ARL13B*), *syntaxin* 19 (*STX19*), and *transmembrane protein 178B* (*TMEM178B*).

Distributions of phenotypes for BW28, BW42, ADFI, RFI, and RFIa by genotype of the most significant SNPs are shown in Figs. 3c–d, 4d–f, and Table 7. These data indicate that the extreme phenotypic values corresponded to the homozygous genotypes, whereas the intermediate values corresponded to the heterozygous genotypes. The genotypic averages (and SE) of the five most significant SNPs on growth and feed efficiency traits are in Table 7. SNPs AX_101003762 and AX_172583407 are closely linked SNPs on GGA28. Broilers that were homozygous *CC* for AX_101003762 and *TT* for AX_172583407 showed significantly larger BW28, BW42, and ADFI than those that were homozygous *TT* and *CC* (*P* < 0.05), respectively. ADFI and RFI were significantly lower for individuals that were homozygous *CC* for AX_75546765 and *CC* for AX_172588157 than those that were homozygous *AA* and *TT* (*P* < 0.01), respectively. The homozygous *AA* individuals for AX_172566874 had lower RFIa, and higher BW42 than those with homozygous *GG* (*P* < 0.05). In addition, these five SNPs had significant additive effects for multiple traits (*P* < 0.05) and did not exhibit significant dominance effects (*P* > 0.05, [see Additional file 11: Table S11]). The effects of the five leading SNPs also differed between male and female broilers [see Additional file 12: Table S12]. Estimates of the additive effects of AX_101003762 on BW28, of AX_75546765 on ADFI, and of AX_172566874 on RFIa were larger for females than for males, while those of AX_172583407 on BW42 and of AX_172588157 on RFI were smaller for females than for males. Except for SNP AX_172583407, the dominance effects of these SNPs were larger for females than for males. These results suggest that the five leading SNPs have additive and pleiotropic effects on growth and feed efficiency traits that are sex-specific.

**Table 7:** Means (+ SE) of growth and feed efficiency traits^a^ by genotype of the five most significant SNPs

SNP (associated trait)	Genotype	BW28 (g)	BW42 (g)	ADFI (g/d)	RFI (g/d)	RFIa (g/d)
AX_101003762 (BW28)	CC (1,825)	1148 ± 7.16^a^	2418 ± 10.81^a^	164.8 ± 0.80^a^	− 0.05 ± 0.44	− 0.02 ± 0.47
	CT (1,234)	1133 ± 6.83^b^	2399 ± 10.28^b^	164.1 ± 0.76^ab^	0.02 ± 0.42	− 0.01 ± 0.46
	TT (255)	1104 ± 8.06^c^	2368 ± 12.19^c^	163.2 ± 0.90^b^	0.28 ± 0.39	0.58 ± 0.43
	*P* value	2.14e−06	9.55e−06	0.03	0.87	0.64
AX_172583407 (BW42)	CC (238)	1107 ± 8.24^c^	2363 ± 12.39^c^	162.3 ± 0.92^c^	− 0.02 ± 0.41	0.00 ± 0.44
	CT (1,222)	1134 ± 7.05^b^	2395 ± 10.57^b^	163.7 ± 0.78^b^	0.03 ± 0.43	0.08 ± 0.47
	TT (1,854)	1146 ± 7.39^a^	2420 ± 11.11^a^	165.1 ± 0.82^a^	− 0.02 ± 0.45	0.00 ± 0.49
	*P* value	1.06e−08	3.64e−13	3.59e−07	0.99	0.97
AX_75546765 (ADFI)	AA (2,414)	1141 ± 13.65	2411 ± 20.48	165.0 ± 1.50^a^	0.27 ± 0.82^a^	0.27 ± 0.91
	AC (841)	1135 ± 13.29	2397 ± 19.93	163.2 ± 1.46^b^	− 0.57 ± 0.80^b^	− 0.49 ± 0.90
	CC (59)	1129 ± 14.16	2374 ± 21.25	159.3 ± 1.56^c^	− 2.9 ± 0.80^c^	− 2.14 ± 0.89
	*P* value	0.39	0.15	2.57e−03	7.40e−03	0.06
AX_172588157 (RFI)	CC (491)	1136 ± 7.00	2397 ± 10.55	162.5 ± 0.77^c^	− 1.26 ± 0.30^c^	− 1.06 ± 0.32^c^
	CT (1,556)	1140 ± 5.42	2408 ± 8.14	164.3 ± 0.60^b^	− 0.13 ± 0.33^b^	− 0.08 ± 0.36^b^
	TT (1,267)	1139 ± 6.44	2410 ± 9.70	165.3 ± 0.71^a^	0.65 ± 0.38^a^	0.60 ± 0.41^a^
	*P* value	0.26	0.06	1.60e−03	1.95e−03	0.02
AX_172566874(RFIa)	AA (681)	1142 ± 6.49	2415 ± 9.79^a^	164.4 ± 0.72	− 0.56 ± 0.25	− 1.13 ± 0.28^c^
	AG (1,634)	1138 ± 4.77	2405 ± 7.17^b^	164.3 ± 0.53	− 0.03 ± 0.29	0.09 ± 0.33^b^
	GG (999)	1139 ± 5.87	2404 ± 8.84^b^	164.6 ± 0.65	0.43 ± 0.35	0.70 ± 0.38^a^
	*P* value	0.32	0.03	0.17	0.14	3.06e−03

^a^BW28, body weight at 28 d of age; BW42, body weight at 42 d of age; ADFI, average daily feed intake; RFI, residual feed intake; RFIa, residual feed intake adjusted for weight of abdominal fat

The evolution in the frequency of the favorable allele of the most significant SNPs over three generations (generation 5, 6, and 7) is shown in Table 8. The frequency of the favorable *C* allele of SNP AX_101003762, which was associated with BW28, increased from 0.71 to 0.78 under selection pressure for increased body weight and growth rate. The frequency of the favorable *T* allele of SNP AX_172583407, which was associated with BW42, increased from 0.72 to 0.78. The frequency of the favorable *A* allele of SNP AX_172566874, which was associated with RFIa, increased from 0.43 to 0.49. The allele frequencies of the other two SNPs were only slightly different between generations.

**Table 8:** Evolution of frequency of the favorable allele of the most significant SNPs in generations 5 to 7

SNPs	Associated traits^a^	Favorable allele	G5^b^	G6	G7
AX_101003762	BW28	C	0.71	0.73	0.78
AX_172583407	BW42	T	0.72	0.76	0.78
AX_75546765	ADFI	C	0.15	0.14	0.14
AX_172588157	RFI	C	0.38	0.39	0.38
AX_172566874	RFIa	A	0.43	0.46	0.49

^a^BW28, body weight at 28 d of age; BW42, body weight at 42 d of age; ADFI, average daily feed intake; RFI, residual feed intake; RFIa, residual feed intake adjusted for weight of abdominal fat

^b^Generation

### Expression of candidate genes in high and low RFI males

**Fig. 5:**
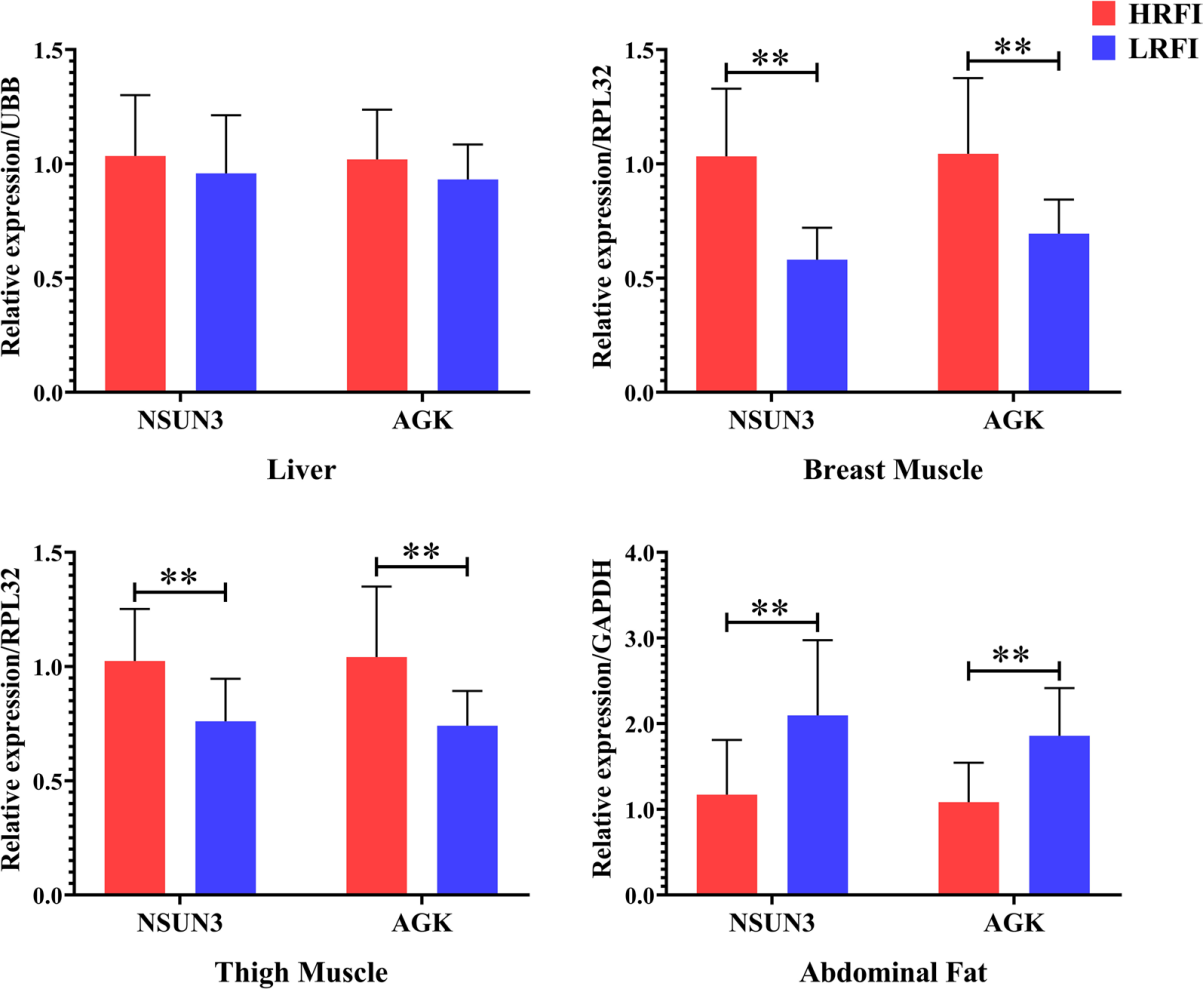
Differentially-expressed genes between the high and low RFI cohorts. Data are expressed as the mean ± SD (n = 15). **P < 0.01

Expression of the three nearest genes (*NSUN3*, *EPHA6*, and *AGK*) within the QTL regions for ADFI, RFI, and RFIa were evaluated by qPCR analysis in males with high and low RFI phenotypes (HRFI and LRFI, Fig. 5). In breast and thigh muscle, the relative expressions of *NSUN3* and *AGK* were significantly higher in HRFI than in LRFI males (*P* < 0.01). In abdominal fat (AbF) tissue, the expressions of *NSUN3* and *AGK* were significantly lower in HRFI than in LRFI individuals (*P* < 0.01). No significant differences between the HRFI and LRFI cohorts were found for the expressions of *NSUN3* and *AGK* in liver. There was no detectable expression of *EPHA6* in the liver, breast muscle, thigh muscle, or AbF tissues.

## Discussion

### Estimates of genetic parameters

The genomic heritability estimates for growth and feed efficiency traits were low to moderate (0.12 to 0.26) and lower than those reported by Bernon and Chambers [37] and Xu et al. [12], who reported pedigree-based estimates that ranged from 0.22 to 0.44 in fast-growing broilers from 28 to 42 days of age, and from 0.22 to 0.56 in medium-growing purebred broilers from 44 to 83 days of age. However, our estimates are consistent with those of Abdollahi-Arpanahi et al. [38], who reported a genomic heritability estimate of 0.23 for BW35 in broilers. Yuan et al. [18] reported low genomic heritability estimates for ADFI (0.15), RFI (0.17), and FCR (0.21) in layer chickens.

Heritability estimates for BW28, BW42, ADFI, RFI, RFIa, ADG, and AbF based on the combined genomic-pedigree relationship matrix were lower than those based on the pedigree relationship matrix. Similar findings were previously reported for pigs [39] and dairy cattle [40, 41]. Several factors can contribute to these differences. First, due to ascertainment bias in the SNP array, particular types of variants, such as rare variants, variants with low MAF, copy number variants, and structural variants may be absent, which can reduce genomic heritabilities. Second, genomic heritabilities and pedigree-based heritabilities apply to different base populations. The pedigree relationship matrix is based on the identity-by-descent (IBD) with respect to a base population consisting of the founders of the pedigree. The genomic relationship matrix is constructed based on allele frequencies in the current population rather than those in the base population because genotypes for previous generations are unknown [25]. The use of the current population results in smaller estimates of additive genetic variance since the current population is expected to be more inbred than the base population [42]. Third, pedigree-based analyses resulted in lower estimates for maternal genetic and maternal environmental variances than analyses using the combined genomic-pedigree relationship matrix for several traits, which may affect estimates of the additive genetic variance [43] and lead to lower genomic heritability estimates. Estimates of genetic correlations between the eight traits were not significantly different based on these two relationship matrices, which is consistent with the result of Abdollahi-Arpanahi et al. [44] in broilers.

The estimate of the genetic correlation between RFI and RFIa was strong (0.83). Similar results for RFI and RFI adjusted for backfat thickness (RFIb) in young beef bulls have been reported by Schenkel et al. [45]. The estimate of heritability was slightly higher for RFI than for RFIa, which is consistent with the results of Ceacero et al. [46], who found slightly higher estimates of heritability for RFI (0.24) than for RFIb (0.20). Our results also indicated that RFI has a moderate genetic correlation with AbF (${\mathrm{r}}_{\mathrm{g}}$ = 0.51), whereas that of RFIa with AbF is close to zero. This suggests that selection for RFI will also result in changes in AbF, while selection for RFIa would change feed intake without affecting AbF. RFIa is usually not a desired trait for a fast-growing broiler breeding program because less abdominal fat weight is preferred. Inclusion of other traits in the model for calculating RFI could be studied in the future.

Sexual dimorphism, largely caused by differences in gene expression [47], has been investigated for economically important traits in livestock species [48]. In the current study, this dimorphism was observed for growth and feed efficiency traits, with males having significantly higher BW28, BW42, ADFI, and ADG than females. In addition, estimates of heritability for growth and feed efficiency traits were lower for males than for females, which is consistent with the results of Mebratie et al. [49] and of van der Heide et al. [48]. Interestingly, the additive effects of the five leading SNPs on growth and feed efficiency traits were also sex-biased, which suggests that the genes located near these SNPs might be differentially expressed between male and female broilers.

### Genome-wide association study of growth and feed efficiency traits

#### Loci and genes for growth traits

A 316.0-kb genomic region on GGA16 was associated with both BW28 and BW42. Previous studies have reported QTL on GGA16 for BW9, BW56, and BW84 in medium-growth broilers [11] and in Iranian indigenous chickens [50]. In the absence of other factors, positive selection will result in a subtle evolution of the favorable allele frequencies over generations. In agreement with this, the frequency of the favorable allele of the most genome-wide significant SNPs (AX_101003762 and AX_172583407) associated with BW28 and BW42 continued to increase from generation 5 to generation 7, because selection for these traits was maintained as the main emphasis. These results substantiate evidence that this region on GGA16 (2.34–2.66 Mb) is associated with BW28 and BW42.

The SNPs, AX_101003762 and AX_172583407, were found to be within the *TRIM39.2* and *ZNF692* genes, respectively. *TRIM39.2* is a member of the RING-B-box-coiled-coil (RBCC)/tripartite motif (TRIM) subfamily of zinc finger proteins that are involved in many biological processes, including cell differentiation [51]. Glycogenin-interacting protein 1 (*GNIP1*) is an isoform of GNIP/TRIM7 and a tripartite motif (TRIM) protein. In vivo overexpression of *GNIP1* in mouse muscle activates the protein kinase B–glycogen synthase kinase-3–glycogen synthase cascade and subsequent glycogen synthesis in muscle, leading to decreased blood glucose levels, lactate levels and mouse body weight, without affecting whole-body insulin or glucose tolerance [52]. ZNF692 was first identified as a transcription factor associated with AMPK signaling [53]. In humans, *ZNF652*, a member of the zinc finger protein gene family is significantly associated with height [54]. Shirai et al. [55] suggested that ZNF692 is a key modulator of hepatic glucose production regulated by AMPK in vivo.

#### Loci and genes for ADFI and RFI

An important region on GGA1 (GGA1: 91.97**–**92.43 Mb) was associated with RFI and a suggestive significant SNP (GGA1: 91.27 Mb) was associated with ADFI. This finding indicates that the 1.16-Mb region (GGA1: 91.27**–**92.43 Mb) is an important QTL for feed efficiency. This region was previously identified as a feed efficiency QTL (GGA1: 90.35**–**123.03 Mb) in a meat-type X egg-type resource chicken population by Hansen et al. [56]. This region contains two candidate genes, namely *NSUN3* and *EPHA6*.

The *NSUN3* gene is a member of the Nol1/Nop2/Sun domain (*NSUN*) family, which is closely related to mitochondrial function and is localized to the mitochondrial (mt) matrix, where it interacts with mt-tRNA^Met^ to methylate cytosine 34 (C34) at the wobble position [57]. A study on *NSUN3*-knockout cells showed strongly decreased mt-tRNA^Met^ methylation and formylation, as well as a reduction in mitochondrial translation, protein synthesis, and reduced oxygen consumption, leading to deficient mitochondrial activity [58, 59]. Recently, Watson et al. [60] showed that *NSUN3* is significantly associated with anorexia nervosa in human. Common symptoms of anorexia nervosa are related to functional impairments of the mitochondrial oxidative phosphorylation system complex I, including poor feeding, neurodegeneration, and muscle weakness [61].

EPHA6 affects physical activity, and thus energy expenditure. *EPHA6* knockout mice display behavioral deficits associated with learning and memory disabilities [62]. Dos Santos et al. [63] found that *EPHA6* was significantly associated with behavioral reactivity in cattle, which is a temperament trait. In humans, *EPHA6* is strongly associated with blood pressure [64]. In addition, Herd and Arthur [65] reported that physical activity accounted for 9% of the phenotypic variation for RFI in beef cattle. As previously reported, the brain plays critical roles in the regulation of feeding behavior and body energy homeostasis [1]. *EPHA6* plays key roles in a variety of biological functions, such as brain development and behavioral regulation. It is highly expressed in most regions of the adult mouse brain, including the hypothalamus [66], which plays a key role in modulating feed intake through the regulation of metabolic hormones and their receptors, such as leptin, neuropeptide Y, agouti-related protein, IGF1, and ghrelin [1, 67].

#### Loci and genes for RFIa

Only one suggestively significant SNP (GGA1: 57.28 Mb) was identified for RFIa. This SNP was located in the first intron of the *AGK* gene. Previous studies found a region on GGA1 (57.0–58.0 Mb) to be significantly associated with FCR in a commercial broiler line and found another SNP, GGaluGA019865, located in the first intron of *AGK*, to be a leading SNP [68]. AGK, also known as multi-substrate lipid kinase (MULK), is abundantly expressed in muscle, heart, kidney, and brain [69, 70]. AGK has lipid kinase-dependent and kinase-independent functions within the mitochondria. On the one hand, by acting as a lipid kinase, it phosphorylates both monoacylglycerol and diacylglycerol to form lysophosphatidic acid (LPA) and phosphatidic acid (PA), respectively [70]. LPA plays an important role in the synthesis of phospholipids and cell proliferation by transactivating the epidermal growth factor (EGF) tyrosine kinase receptor [71]. PA acts both as an essential molecule for mitochondrial ultrastructure and function, and as a second messenger that regulates numerous biological processes, including activation of the mammalian target of the rapamycin (mTOR) signaling pathway [72, 73]. On the other hand, AGK is a subunit of the inner membrane 22 (TIM22) complex, which imports and assembles mitochondrial carrier proteins [74, 75]. Inactivation of *AGK* leads to the reduction of mitochondrial maximal respiration and tricarboxylic acid (TCA) cycle flux in cells [74]. In summary, the *NSUN3* and *AGK* genes play an important role in the regulation of the mitochondrial function.

Mitochondrial function has previously been associated with feed efficiency in poultry and livestock, because mitochondria are responsible for producing approximately 90% of the energy of the cells [76]. In our study, the lower expression of *NSUN3* and *AGK* in breast and thigh muscle of LRFI chickens may result from decreased mitochondrial energy metabolism, which is consistent with previous observations in the skeletal muscle of pigs from LRFI and HRFI lines [77]. It is possible that the significant SNPs identified in this study are in LD with the causative mutations. However, additional strategies are needed in the future for fine mapping the causal mutation.

## Conclusions

Heritability estimates for growth and feed efficiency traits in broilers from 28 to 41 days of age were low to moderate, ranging from 0.12 to 0.26. Narrow QTL regions with additive pleiotropic effects were associated with BW28 and BW42 (spanning 0.32 Mb) and RFI (spanning 1.16 Mb). The most likely candidate genes for these QTL, *NSUN3*, *EPHA6*, and *AGK*, are known to be involved in mitochondrial function and behavioral regulation. These results contribute to the identification of candidate genes and SNPs for feed efficiency traits in poultry.

## Supplementary Information

**Additional file 1: Table S1.** Statistics of selection pressure for males and females.

**Additional file 2: Table S2.** Statistics of the number of male and female broilers, separately, slaughtered in hatches and generations 5 to 7.

**Additional file 3: Table S3.** Distribution of SNPs after quality control.

**Additional file 4: Table S4.** Primer sequences for qPCR analysis.

**Additional file 5: Table S5.** Phenotypes for individual broilers.

**Additional file 6: Table S6.** Descriptive statistics for male and female broilers.

**Additional file 7: Table S7.** Estimates of variance components and heritabilities for males and females using the relationship matrix that blends genomic and pedigree information.

**Additional file 8: Table S8.** Estimates of variance components and heritabilities for males and females using the pedigree relationship matrix.

**Additional file 9: Table S9.** Estimates of genetic and phenotypic correlations among growth and feed efficiency traits based on the pedigree relationship matrix.

**Additional file 10: Table S10.** Sequences of SNPs reported in Tables 4 and 5.

**Additional file 11: Table S11.** Additive and dominance effects of the five most significant SNPs on growth and feed efficiency traits.

**Additional file 12: Table S12.** Means (+ SE) of growth and feed efficiency traits by genotype of the five most significant SNPs in males and females.

## Data Availability

Data sharing is not applicable to this article since no datasets were generated or analyzed during the current study.
