# Supplementary material for: Identification of QTL regions and candidate genes for growth and feed efficiency in broilers
Source: Genet Sel Evol. 2021 Feb 6;53:13. doi: 10.1186/s12711-021-00608-3 (PMC7866652; doi:10.1186/s12711-021-00608-3)
Supplement: Supplementary file 1 — Additional file 1: Table S1. Statistics of selection pressure for males and females. [file 12711_2021_608_MOESM1_ESM.docx]

**Table S1 Statistics of selection pressure for males and females**

| **Generation^a^** | **Males** | | | **Females** | | |
| --- | --- | --- | --- | --- | --- | --- |
|  | **N1^b^** | **N2^c^** | **SP^d^** | **N1** | **N2** | **SP** |
| G2 | 3,298 | 80 | 0.024 | 3,218 | 810 | 0.252 |
| G3 | 2,957 | 80 | 0.027 | 3,413 | 800 | 0.234 |
| G4 | 4,033 | 80 | 0.020 | 4,276 | 960 | 0.225 |
| G5 | 3,656 | 80 | 0.022 | 3,893 | 960 | 0.247 |
| G6 | 3,099 | 76 | 0.025 | 3,215 | 912 | 0.284 |
| G7 | 2,566 | 120 | 0.047 | 2,763 | 960 | 0.347 |

^a^2,502 broilers (1571 from G5, 446 from G6, and 485 from G7) slaughtered at 42 d of age did not included.

^b^N1, the number of chickens before selection.

^c^N2, the number of chickens after selection.

^d^SP, selection pressure, which is calculated by N2 divided by N1.
