# Supplementary material for: Identification of QTL regions and candidate genes for growth and feed efficiency in broilers
Source: Genet Sel Evol. 2021 Feb 6;53:13. doi: 10.1186/s12711-021-00608-3 (PMC7866652; doi:10.1186/s12711-021-00608-3)
Supplement: Supplementary file 2 — Additional file 2: Table S2. Statistics of the number of male and female broilers, separately, slaughtered in hatches and generations 5 to 7. [file 12711_2021_608_MOESM2_ESM.docx]

**Table S2 Statistics of the number of male and female broilers slaughtered in hatches** **and generations 5 to 7**

| **Generation** | **Hatch** | **Number of males** | **Number of females** | **Number of males slaughtered** | **Number of females slaughtered** |
| --- | --- | --- | --- | --- | --- |
| G5 | 1 | 323 | 376 | 323 | 376 |
|  | 2 | 407 | 465 | 407 | 465 |
|  | 3 | 105 | — | — | — |
|  | 4 | 154 | — | — | — |
| G6 | 5 | 178 | 268 | 178 | 268 |
|  | 6 | 53 | — | — | — |
|  | 7 | 62 | — | — | — |
|  | 8 | 44 | — | — | — |
| G7 | 9 | 229 | 256 | 229 | 256 |
|  | 10 | 308 | — | — | — |
|  | 11 | 137 | — | — | — |
