# Supplementary material for: Identification of QTL regions and candidate genes for growth and feed efficiency in broilers
Source: Genet Sel Evol. 2021 Feb 6;53:13. doi: 10.1186/s12711-021-00608-3 (PMC7866652; doi:10.1186/s12711-021-00608-3)
Supplement: Supplementary file 3 — Additional file 3: Table S3. Distribution of SNPs after quality control. [file 12711_2021_608_MOESM3_ESM.docx]

**Table S3 Distribution of SNPs after quality control**

| **GGA^a^** | **Physical Map (Mb)^b^** | **No. of SNP markers** | **Average distance (kb)** |
| --- | --- | --- | --- |
| 1 | 197.68 | 8,069 | 24.50 |
| 2 | 149.68 | 5,679 | 26.36 |
| 3 | 110.85 | 4,250 | 26.08 |
| 4 | 91.32 | 3,672 | 24.87 |
| 5 | 59.81 | 2,171 | 27.55 |
| 6 | 36.43 | 1,436 | 25.37 |
| 7 | 36.74 | 1,400 | 26.24 |
| 8 | 30.22 | 1,082 | 27.93 |
| 9 | 24.18 | 1,019 | 23.73 |
| 10 | 21.12 | 1,162 | 18.18 |
| 11 | 20.20 | 1,101 | 18.35 |
| 12 | 20.39 | 1,124 | 18.14 |
| 13 | 19.17 | 843 | 22.74 |
| 14 | 16.22 | 871 | 18.62 |
| 15 | 13.06 | 606 | 21.56 |
| 16 | 2.99 | 48 | 62.38 |
| 17 | 10.76 | 591 | 18.21 |
| 18 | 11.37 | 553 | 20.57 |
| 19 | 10.32 | 585 | 17.65 |
| 20 | 13.90 | 716 | 19.41 |
| 21 | 6.84 | 398 | 17.20 |
| 22 | 5.46 | 90 | 60.67 |
| 23 | 6.20 | 252 | 24.61 |
| 24 | 6.49 | 298 | 21.78 |
| 25 | 3.98 | 73 | 54.55 |
| 26 | 6.06 | 272 | 22.26 |
| 27 | 8.08 | 198 | 40.81 |
| 28 | 5.12 | 218 | 23.48 |
| Z | 82.55 | 2,387 | 34.58 |

^a^*Gallus gallus* chromosome.

^b^Physical length of the chromosome based on Gallus gallus-6.0.
