# Supplementary material for: Identification of QTL regions and candidate genes for growth and feed efficiency in broilers
Source: Genet Sel Evol. 2021 Feb 6;53:13. doi: 10.1186/s12711-021-00608-3 (PMC7866652; doi:10.1186/s12711-021-00608-3)
Supplement: Supplementary file 4 — Additional file 4: Table S4. Primer sequences for qPCR analysis. [file 12711_2021_608_MOESM4_ESM.docx]

**Table S4** **Primer sequences for qPCR**

| **Gene name** | **Forward primer (5’ to 3’)** | **Reversed primer (5’ to 3’)** | **Product length (bp)** | **GenBank No.** |
| --- | --- | --- | --- | --- |
| *NSUN3* | GCTCAATGCTGCTTCTGTGTTACC | GTTCTCCAATCTGTCTACCGTCCA | 254 | XM_416643.6 |
| *EPHA6* | TCAGGACGGCAGCAGGATACA | AGTGAGGATGACCAGGAGAGTGAA | 248 | XM_015297990.2 |
| *AGK* | GCACCTGTCTGGCTTGGATGTAA | CCTCATCTGCTCTGCGAAGAAGTC | 161 | XM_004937875.3 |
| *RPL32* | CCGCCACCAGTCTGATCGCTAT | GCTTCGTCTTCTTGTTGCTCCCAT | 140 | NM_001252255.1 |
| *UBB* | CCCAGTGATACCATCGAGAAT | ACAAAGATCTGCATGCCAC | 188 | NM_001293174.1 |
| *GAPDH* | AGAACATCATCCCAGCGTCCACT | CGGCAGGTCAGGTCAACAACAG | 133 | NM_204305.1 |
