# Supplementary material for: Identification of QTL regions and candidate genes for growth and feed efficiency in broilers
Source: Genet Sel Evol. 2021 Feb 6;53:13. doi: 10.1186/s12711-021-00608-3 (PMC7866652; doi:10.1186/s12711-021-00608-3)
Supplement: Supplementary file 6 — Additional file 6: Table S6. Descriptive statistics for male and female broilers. [file 12711_2021_608_MOESM6_ESM.docx]

**Table S****6** **Descriptive statistics for male and female broilers**

| **Traits^a^** | **Sex** | **N** | **Mean** | **SD** | **Min** | **Max** | **CV (%)** |
| --- | --- | --- | --- | --- | --- | --- | --- |
| BW28 (g) | Male | 1,972 | 1,131^**^ | 169 | 557 | 1,530 | 14.98 |
|  | Female | 1,342 | 1,001 | 156 | 569 | 1,400 | 15.61 |
| BW42 (g) | Male | 1,972 | 2,360^**^ | 332 | 1,327 | 3,120 | 14.07 |
|  | Female | 1,342 | 1,980 | 285 | 1,198 | 2,845 | 14.39 |
| ADFI (g/d) | Male | 1,972 | 160.0^**^ | 23.7 | 92.4 | 222.0 | 14.83 |
|  | Female | 1,342 | 138.4 | 22.2 | 88.2 | 208.9 | 16.04 |
| RFI (g/d) | Male | 1,972 | 0.00 | 5.89 | -19.33 | 19.03 | — |
|  | Female | 1,342 | 0.00 | 6.17 | -18.79 | 18.86 | — |
| RFIa (g/d) | Male | 1,117 | 0.00 | 6.01 | -17.99 | 16.98 | — |
|  | Female | 1,336 | 0.00 | 5.70 | -17.45 | 18.13 | — |
| ADG (g/d) | Male | 1,972 | 87.82^**^ | 14.31 | 45.36 | 127.86 | 16.29 |
|  | Female | 1,342 | 69.89 | 10.87 | 40.43 | 105.00 | 15.56 |
| FCR (g/g) | Male | 1,972 | 1.83 | 0.12 | 1.43 | 2.38 | 6.79 |
|  | Female | 1,342 | 1.98^**^ | 0.13 | 1.58 | 2.39 | 6.52 |
| AbF (g) | Male | 1,117 | 33.41 | 11.14 | 3.30 | 90.80 | 33.34 |
|  | Female | 1,336 | 35.84^**^ | 11.08 | 1.20 | 82.40 | 30.93 |

^a^BW28, body weight at 28 d of age; BW42, body weight at 42 d of age; ADFI, average daily feed intake; RFI, residual feed intake; RFIa, residual feed intake adjusted for weight of abdominal fat; ADG, average daily gain; FCR, feed conversion ratio; AbF, weight of abdominal fat; CV, coefficient of variation; ***P* < 0.01.
