# Supplementary material for: Identification of QTL regions and candidate genes for growth and feed efficiency in broilers
Source: Genet Sel Evol. 2021 Feb 6;53:13. doi: 10.1186/s12711-021-00608-3 (PMC7866652; doi:10.1186/s12711-021-00608-3)
Supplement: Supplementary file 7 — Additional file 7: Table S7. Estimates of variance components and heritabilities for males and females using the relationship matrix that blends genomic and pedigree information. [file 12711_2021_608_MOESM7_ESM.docx]

**Table S7** **Estimates of variance components and heritabilities for males and females using the relationship matrix that blends genomic and pedigree information**

| **Traits^a^** | **Gender** | **Parameter^b^** | | | | | | | |
| --- | --- | --- | --- | --- | --- | --- | --- | --- | --- |
|  |  | $\boldsymbol{\sigma}_{\mathbf{a}}^{\mathbf{2}}$ | $\boldsymbol{\sigma}_{\mathbf{m}}^{\mathbf{2}}$ | $\boldsymbol{\sigma}_{\mathbf{am}}$ | $\boldsymbol{\sigma}_{\mathbf{c}}^{\mathbf{2}}$ | $\boldsymbol{\sigma}_{\mathbf{e}}^{\mathbf{2}}$ | $\mathbf{h}_{\mathbf{a}}^{\mathbf{2}}$ | $\mathbf{h}_{\mathbf{m}}^{\mathbf{2}}$ | **c^2^** |
| BW28 | Male | 1,240±393 | 749.2±509.6 | -364.0±401.7 | — | 6,717±400 | 0.15±0.05 | 0.09±0.06 | — |
|  | Female | 2,993±781 | 1,604±1,149 | -724.0±849.3 | — | 7,008±702 | 0.28±0.07 | 0.15±0.10 | — |
| BW42 | Male | 3,755±1,047 | 829.3±1,088.0 | -749.7±953.7 | — | 16,253±1,001 | 0.19±0.05 | 0.04±0.05 | — |
|  | Female | 9,172±2,030 | 4,507±2,610 | -4,682±2,107 | — | 14,799±1,655 | 0.39±0.08 | 0.19±0.11 | — |
| ADFI | Male | 25.66±6.77 | 5.29±6.08 | -7.52±5.86 | — | 98.03±6.23 | 0.21±0.05 | 0.04±0.05 | — |
|  | Female | 49.03±10.00 | 22.38±11.30 | -27.30±9.62 | — | 63.01±7.77 | 0.46±0.09 | 0.21±0.10 | — |
| RFI | Male | 6.36±1.81 | 1.67±1.84 | -1.80±1.63 | 0.22±1.44 | 27.74±1.73 | 0.19±0.05 | 0.05±0.05 | 0.01±0.04 |
|  | Female | 12.26±3.00 | 4.29±2.69 | -3.30±2.80 | — | 23.85±2.34 | 0.33±0.08 | 0.12±0.07 | — |
| RFIa | Male | 4.57±2.67 | 3.27±3.68 | -3.85±2.89 | 1.62±2.63 | 30.38±2.81 | 0.13±0.07 | 0.09±0.10 | 0.04±0.07 |
|  | Female | 7.10±2.25 | 1.66±2.18 | -1.21±2.16 | — | 23.99±2.03 | 0.23±0.07 | 0.05±0.07 | — |
| ADG | Male | 6.20±1.87 | — | — | 0.28±1.64 | 44.22±2.44 | 0.12±0.04 | — | 0.01±0.03 |
|  | Female | 12.98±3.32 | 6.97±4.03 | -8.23±3.36 | — | 27.72±2.85 | 0.33±0.08 | 0.18±0.10 | — |
| FCR | Male | 0.0008±0.0003 | — | — | — | 0.0089±0.0004 | 0.08±0.03 | — | — |
|  | Female | 0.0024±0.0008 | 0.0010±0.0007 | — | — | 0.0117±0.0009 | 0.16±0.05 | 0.06±0.05 | — |
| AbF | Male | 18.71±5.75 | 2.71±6.31 | — | — | 65.13±6.10 | 0.22±0.06 | 0.03±0.07 | — |
|  | Female | 49.03±8.80 | 26.01±9.99 | -26.90±8.56 | — | 44.96±6.36 | 0.53±0.09 | 0.28±0.10 | — |

^a^BW28, body weight at 28 d of age; BW42, body weight at 42 d of age; ADFI, average daily feed intake; RFI, residual feed intake; RFIa, residual feed intake adjusted for weight of abdominal fat; ADG, average daily gain; FCR, feed conversion ratio; AbF, weight of abdominal fat.

^b^$\sigma_{a}^{2}$, direct additive genetic variance; $\sigma_{m}^{2}$, maternal additive genetic variance; $\sigma_{\mathrm{am}}$, covariance between direct and maternal genetic effects; $\sigma_{c}^{2}$, common maternal environment variance;$\sigma_{e}^{2}$, residual error variance; $h_{a}^{2}$, direct heritability; $h_{m}^{2}$, maternal heritability; c^2^, maternal environmental variance as a proportion of phenotypic variance. — represents close to zero.
