# Supplementary material for: Identification of QTL regions and candidate genes for growth and feed efficiency in broilers
Source: Genet Sel Evol. 2021 Feb 6;53:13. doi: 10.1186/s12711-021-00608-3 (PMC7866652; doi:10.1186/s12711-021-00608-3)
Supplement: Supplementary file 8 — Additional file 8: Table S8. Estimates of variance components and heritabilities for males and females using the pedigree relationship matrix. [file 12711_2021_608_MOESM8_ESM.docx]

**Table S8** **Estimates of variance components and heritabilities for males and females using the pedigree relationship matrix**

| **Traits^a^** | **Gender** | **Parameter^b^** | | | | | | | |
| --- | --- | --- | --- | --- | --- | --- | --- | --- | --- |
|  |  | $\boldsymbol{\sigma}_{\mathbf{a}}^{\mathbf{2}}$ | $\boldsymbol{\sigma}_{\mathbf{m}}^{\mathbf{2}}$ | $\boldsymbol{\sigma}_{\mathbf{am}}$ | $\boldsymbol{\sigma}_{\mathbf{c}}^{\mathbf{2}}$ | $\boldsymbol{\sigma}_{\mathbf{e}}^{\mathbf{2}}$ | $\mathbf{h}_{\mathbf{a}}^{\mathbf{2}}$ | $\mathbf{h}_{\mathbf{m}}^{\mathbf{2}}$ | **c^2^** |
| BW28 | Male | 2,109±665 | 552.7±531.9 | -673.0±519.3 | — | 6,447±481 | 0.25±0.08 | 0.07±0.06 | — |
|  | Female | 3,918±1,033 | — | — | 344.7±587.8 | 6,872±783 | 0.35±0.09 | — | 0.03±0.05 |
| BW42 | Male | 5,009±1,581 | 1,017±989 | -1,696±1,149 | — | 15,938±1,102 | 0.25±0.08 | 0.05±0.05 | — |
|  | Female | 9,169±2,340 | — | — | — | 15,531±1,756 | 0.37±0.09 | — | — |
| ADFI | Male | 25.89±8.80 | 4.81±5.69 | -8.18±6.50 | — | 99.68±6.47 | 0.21±0.07 | 0.04±0.05 | — |
|  | Female | 32.55±9.24 | — | — | — | 78.05±7.52 | 0.29±0.08 | — | — |
| RFI | Male | 6.80±2.47 | 1.01±1.93 | -1.53±1.93 | — | 28.15±1.91 | 0.20±0.07 | 0.03±0.06 | — |
|  | Female | 14.09±4.57 | 4.30±4.00 | -5.66±3.64 | — | 25.43±3.12 | 0.37±0.11 | 0.11±0.10 | — |
| RFIa | Male | 5.18±3.64 | 3.25±4.10 | -3.33±3.52 | 0.62±2.75 | 30.67±2.97 | 0.14±0.10 | 0.09±0.11 | 0.02±0.08 |
|  | Female | 8.47±3.35 | 2.28±2.60 | -3.06±2.67 | — | 24.49±2.35 | 0.26±0.10 | 0.07±0.08 | — |
| ADG | Male | 4.31±2.61 | 0.12±2.34 | -0.68±2.10 | 0.81±2.14 | 46.02±2.58 | 0.09±0.05 | 0.00±0.05 | 0.02±0.04 |
|  | Female | 9.62±2.77 | — | — | — | 30.75±2.65 | 0.24±0.07 | — | — |
| FCR | Male | 0.0007±0.0003 | — | — | — | 0.0090±0.0004 | 0.07±0.04 | — | — |
|  | Female | 0.0032±0.0011 | — | — | 0.0003±0.0008 | 0.0116±0.0010 | 0.21±0.07 | — | 0.02±0.05 |
| AbF | Male | 23.84±7.86 | — | — | — | 63.32±6.58 | 0.27±0.09 | — | — |
|  | Female | 61.94±14.99 | 36.91±12.96 | -42.42±12.32 | — | 42.89±8.92 | 0.62±0.14 | 0.37±0.12 | — |

^a^BW28, body weight at 28 d of age; BW42, body weight at 42 d of age; ADFI, average daily feed intake; RFI, residual feed intake; RFIa, residual feed intake adjusted for weight of abdominal fat; ADG, average daily gain; FCR, feed conversion ratio; AbF, weight of abdominal fat.

^b^$\sigma_{a}^{2}$, direct additive genetic variance; $\sigma_{m}^{2}$, maternal additive genetic variance; $\sigma_{\mathrm{am}}$, covariance between direct and maternal genetic effects; $\sigma_{c}^{2}$, common maternal environment variance;$\sigma_{e}^{2}$, residual error variance; $h_{a}^{2}$, direct heritability; $h_{m}^{2}$, maternal heritability; c^2^, maternal environmental variance as a proportion of phenotypic variance. — represents close to zero.
