# Supplementary material for: Identification of QTL regions and candidate genes for growth and feed efficiency in broilers
Source: Genet Sel Evol. 2021 Feb 6;53:13. doi: 10.1186/s12711-021-00608-3 (PMC7866652; doi:10.1186/s12711-021-00608-3)
Supplement: Supplementary file 9 — Additional file 9: Table S9. Estimates of genetic and phenotypic correlations among growth and feed efficiency traits based on the pedigree relationship matrix. [file 12711_2021_608_MOESM9_ESM.docx]

**Table S9** **Estimates of genetic and phenotypic correlations among growth and feed efficiency traits** **based on the pedigree relationship matrix**

| **Traits^a^** | **BW28** | **BW42** | **ADFI** | **RFI** | **RFIa** | **ADG** | **FCR** | **AbF** |
| --- | --- | --- | --- | --- | --- | --- | --- | --- |
| **BW28** |  | 0.90±0.03 | 0.56±0.09 | -0.13±0.13 | -0.05±0.16 | 0.44±0.12 | 0.22±0.14 | 0.35±0.10 |
| **BW42** | 0.77±0.01 |  | 0.80±0.05 | 0.02±0.13 | 0.01±0.16 | 0.79±0.05 | -0.02±0.15 | 0.36±0.10 |
| **ADFI** | 0.41±0.02 | 0.78±0.01 |  | 0.57±0.1 | 0.38±0.15 | 0.86±0.04 | 0.16±0.15 | 0.48±0.10 |
| **RFI** | -0.02±0.02 | -0.01±0.02 | 0.54±0.01 |  | 0.79±0.05 | 0.19±0.15 | 0.65±0.09 | 0.42±0.12 |
| **RFIa** | -0.02±0.02 | -0.01±0.02 | 0.47±0.02 | 0.92±0.00 |  | 0.11±0.19 | 0.47±0.14 | -0.02±0.16 |
| **ADG** | 0.17±0.02 | 0.76±0.01 | 0.79±0.01 | 0.00±0.02 | 0.00±0.02 |  | -0.35±0.14 | 0.24±0.13 |
| **FCR** | 0.27±0.02 | -0.18±0.02 | 0.05±0.02 | 0.71±0.01 | 0.61±0.01 | -0.55±0.01 |  | 0.35±0.13 |
| **AbF** | 0.29±0.02 | 0.35±0.02 | 0.46±0.02 | 0.32±0.02 | -0.01±0.02 | 0.26±0.02 | 0.17±0.02 |  |

^a^Upper diagonal is genetic correlation, and lower diagonal is phenotypic correlation; BW28, body weight at 28 d of age; BW42, body weight at 42 d of age; ADFI, average daily feed intake; RFI, residual feed intake; RFIa, residual feed intake adjusted for weight of abdominal fat; ADG, average daily gain; FCR, feed conversion ratio; AbF, weight of abdominal fat.
