# Supplementary material for: Identification of QTL regions and candidate genes for growth and feed efficiency in broilers
Source: Genet Sel Evol. 2021 Feb 6;53:13. doi: 10.1186/s12711-021-00608-3 (PMC7866652; doi:10.1186/s12711-021-00608-3)
Supplement: Supplementary file 11 — Additional file 11: Table S11. Additive and dominance effects of the five most significant SNPs on growth and feed efficiency traits. [file 12711_2021_608_MOESM11_ESM.docx]

**Table S11** **Additive and dominance effects of the five most significant SNPs on growth and feed efficiency traits^a^**

| **SNP (****associated trait)** | **Effect^b^** | **BW28 (g)** | **BW42 (g)** | **ADFI (g/d)** | **RFI (g/d)** | **RFIa (g/d)** |
| --- | --- | --- | --- | --- | --- | --- |
| AX_101003762  (BW28) | Additive | 22.05±3.58^**^ | 24.94±5.40^**^ | 0.80±0.40^*^ | -0.16±0.22 | -0.30±0.24 |
|  | Dominance | 6.50±4.27 | 6.41±6.41 | 0.12±0.47 | -0.10±0.26 | -0.29±0.29 |
| AX_172583407  (BW42) | Additive | 19.89±3.70^**^ | 28.71±5.55^**^ | 1.39±0.41^**^ | 0.00±0.22 | 0.00±0.24 |
|  | Dominance | 6.93±4.35 | 3.13±6.52 | -0.04±0.48 | 0.05±0.26 | 0.08±0.29 |
| AX_75546765  (ADFI) | Additive | 5.88±6.82 | 18.74±10.24^*^ | 2.84±0.75^**^ | 1.59±0.41^**^ | 1.21±0.45^**^ |
|  | Dominance | 0.61±7.24 | 4.71±10.85 | 1.10±0.80 | 0.75±0.44 | 0.45±0.49 |
| AX_172588157  (RFI) | Additive | 1.33±3.22 | 6.57±4.85 | 1.44±0.35^**^ | 0.96±0.19^**^ | 0.83±0.21^**^ |
|  | Dominance | 3.01±3.60 | 4.30±5.40 | 0.37±0.40 | 0.17±0.22 | 0.15±0.25 |
| AX_172566874  (RFIa) | Additive | -1.93±2.94 | -5.38±4.42 | 0.14±0.33 | 0.50±0.18^**^ | 0.92±0.19^**^ |
|  | Dominance | -2.72±3.43 | -4.05±5.14 | -0.18±0.38 | 0.04±0.21 | 0.30±0.24 |

^a^BW28, body weight at 28 d of age; BW42, body weight at 42 d of age; ADFI, average daily feed intake; RFI, residual feed intake; RFIa, residual feed intake adjusted for weight of abdominal fat.

^b^The additive effect (*a*) was estimated using the formula *a* = (CC-TT)/2; The dominance effect (*d*) was estimated using the formula *d* = CT-[(CC+TT)/2]. ***P* < 0.01; **P* < 0.05.
