# Supplementary material for: Identification of QTL regions and candidate genes for growth and feed efficiency in broilers
Source: Genet Sel Evol. 2021 Feb 6;53:13. doi: 10.1186/s12711-021-00608-3 (PMC7866652; doi:10.1186/s12711-021-00608-3)
Supplement: Supplementary file 12 — Additional file 12: Table S12. Means (+ SE) of growth and feed efficiency traits by genotype of the five most significant SNPs in males and females. [file 12711_2021_608_MOESM12_ESM.docx]

**Table S12** **Means (+SE) of growth and feed efficiency traits by genotype of the five most significant SNPs in males and females**

| **SNP/Trait^a^** | **Genotype** | **Males** | | **Females** | |
| --- | --- | --- | --- | --- | --- |
|  |  | ***N*** | **LSM±SE** | ***N*** | **LSM±SE** |
| AX_101003762  BW28 (g) | CC | 1,113 | 1187±8.79^a^ | 712 | 1040±11.05^a^ |
|  | CT | 716 | 1173±8.63^b^ | 518 | 1024±10.85^b^ |
|  | TT | 143 | 1150±9.79^c^ | 112 | 986±11.44^c^ |
| Additive effect |  |  | 18.83±4.40^**^ |  | 27.11±5.53^**^ |
| Dominance effect |  |  | 4.37±5.40 |  | 11.74±6.89 |
| AX_172583407  BW42 (g) | CC | 122 | 2440±15.61^c^ | 116 | 2018±16.85^c^ |
|  | CT | 701 | 2490±14.09^b^ | 521 | 2024±15.76^b^ |
|  | TT | 1,149 | 2517±14.38^a^ | 705 | 2049±16.35^a^ |
| Additive effect |  |  | 38.45±7.19^**^ |  | 15.57±8.17^**^ |
| Dominance effect |  |  | 11.11±8.61 |  | -8.99±9.97 |
| AX_75546765  ADFI (g/d) | AA | 1,424 | 171.2±1.90^a^ | 990 | 143.8±2.26^a^ |
|  | AC | 511 | 169.6±1.89^a^ | 330 | 142.0±2.24^a^ |
|  | CC | 37 | 167.1±1.98^b^ | 22 | 134.2±2.29^b^ |
| Additive effect |  |  | 2.04±0.95^**^ |  | 4.79±1.13^**^ |
| Dominance effect |  |  | 0.49±1.03 |  | 2.98±1.23^*^ |
| AX_172588157  RFI (g/d) | CC | 491 | -1.45±0.36^c^ | 200 | -1.00±0.46 |
|  | CT | 1,556 | -0.24±0.4^b^ | 607 | 0.00±0.51 |
|  | TT | 1,267 | 0.80±0.46^a^ | 535 | 0.27±0.58 |
| Additive effect |  |  | 1.12±0.23^**^ |  | 0.64±0.29^*^ |
| Dominance effect |  |  | 0.09±0.28 |  | 0.37±0.35 |
| AX_172566874  RFIa (g/d) | AA | 220 | -0.71±0.41 | 255 | -1.51±0.37^c^ |
|  | AG | 557 | 0.03±0.48 | 658 | 0.13±0.43^b^ |
|  | GG | 340 | 0.40±0.55 | 423 | 0.78±0.50^a^ |
| Additive effect |  |  | 0.55±0.27^*^ |  | 1.14±0.25^**^ |
| Dominance effect |  |  | 0.18±0.36 |  | 0.49±0.31 |

^a^BW28, body weight at 28 d of age; BW42, body weight at 42 d of age; ADFI, average daily feed intake; RFI, residual feed intake; RFIa, residual feed intake adjusted for weight of abdominal fat. The additive effect (*a*) was estimated using the formula *a* = (CC-TT)/2; The dominance effect (*d*) was estimated using the formula *d* = CT-[(CC+TT)/2]. ^a-c^Means within columns with different lowercase superscript letters were significantly different (*P* < 0.05). ***P* < 0.01; **P* < 0.05.
